# Supplementary material for: Ce-induced synergistic effect in exsolved perovskite catalyst for highly efficient and robust methane dry reforming
Source: Nat Commun. 2025 Nov 27;16:10630. doi: 10.1038/s41467-025-65619-w (PMC12660910; doi:10.1038/s41467-025-65619-w)
Supplement: Supplementary file 1 — Supplementary Information [file 41467_2025_65619_MOESM1_ESM.pdf]

Supplementary Information

Ce-induced synergistic effect in exsolved perovskite catalyst for highly efficient and robust methane dry reforming

Chencun Hao [a], Zhiyu Qu [b], Louise R. Smith [c], Nicholas F. Dummer [c], Haifeng Qi [c], Thomas J.A. Slater [c], Zhiping Zhu [d], Riguang Zhang [b*], Zhao Sun [ac*], Zhiqiang Sun [a*], Graham J. Hutchings [c*]

[a] Hunan Engineering Research Center of Clean and Low-Carbon Energy Technology, School of Energy Science and Engineering, Central South University, Changsha 410083, China

[b] State Key Laboratory of Clean and Efficient Coal Utilization, College of Chemical Engineering and Technology, Taiyuan University of Technology, Taiyuan, Shanxi 030024, China

[c] Max Planck-Cardiff Centre on the Fundamentals of Heterogeneous Catalysis FUNCAT, Cardiff Catalysis Institute, School of Chemistry, Cardiff University, Cardiff CF24 4HQ, United Kingdom.

[d] State Key Laboratory of Coal Conversion, Institute of Engineering Thermophysics, Chinese Academy of Sciences, Beijing 100190, China

Emails

zhangriguang@tyut.edu.cn; zhaosun@csu.edu.cn; zqsun@csu.edu.cn; Hutch@cardiff.ac.uk

Chart List

Figure S1. The conversion rates of CH_4 and CO_2, selectivity of H_2 and CO, and H_2/CO ratio at different temperatures for sample R-0.2Ce at WHSV=24000 $mL\cdot g^{-1}\cdot h^{-1}$.

Figure S2. The conversion rates of CH_4 and CO_2, selectivity of H_2 and CO, and H_2/CO ratio at different temperatures for sample R-0Ce at WHSV=12000 $mL\cdot g^{-1}\cdot h^{-1}$.

Figure S3. The conversion rates of CH_4 and CO_2, selectivity of H_2 and CO, and H_2/CO ratio at different temperatures for sample R-0.4Ce at WHSV=12000 $mL\cdot g^{-1}\cdot h^{-1}$.

Figure S4. CH_4 and CO_2 conversion rate of stability test of R-0.2Ce and R-0Ce for 120 h and 60 h. Evaluated conditions: 750 °C, CH_4: CO_2: N_2=1: 1: 2, WHSV=12000 $mL\cdot g^{-1}\cdot h^{-1}$.

Figure S5. H_2/CO value of stability test of R-0.2Ce and R-0Ce for 120 h and 60 h. Evaluated conditions: 750 °C, CH_4: CO_2: N_2=1: 1:2, WHSV=12000 $mL\cdot g^{-1}\cdot h^{-1}$.

Figure S6. XRD patterns of fresh, 1h DRM reacted, and 3h DRM reacted 0.2Ce-$La_{0.97}Ni_{0.4}Cr_{0.6}O_3$.

Figure S7. SEM images of fresh 0.2Ce-$La_{0.97}Ni_{0.4}Cr_{0.6}O_3$.

Figure S8. SEM images of 1 h DRM reacted 0.2Ce-$La_{0.97}Ni_{0.4}Cr_{0.6}O_3$.

Figure S9. SEM images of 3 h DRM reacted 0.2Ce-$La_{0.97}Ni_{0.4}Cr_{0.6}O_3$.

Figure S10. Particle size and density distribution of R-0.2Ce sample with different DRM reaction time (Obtained by ImageJ software through SEM image statistics).

Figure S11. **a** Ce 3*d*, **b** Cr 2*p*, **c** Ni 3*p*, **d** O 1*s* XPS spectra of fresh 0.2Ce-$La_{0.97}Ni_{0.4}Cr_{0.6}O_3$, after 1h DRM reacted, and after 3h DRM reacted.

Figure S12. The H_2/CO value of R-0.2Ce and R-0Ce in the DRM stability test.

Figure S13. XRD patterns of calcined $LaCrO_3$, $LaNi_{0.4}Cr_{0.6}O_3$, and *x*Ce-$La_{0.97}Ni_{0.4}Cr_{0.6}O_3$.

Figure S14. TEM images and EDX mapping of $LaNi_{0.4}Cr_{0.6}O_3$ after 800 °C, 4 h calcined.

Figure S15. TEM images and EDX mapping of 0.2Ce-$La_{0.97}Ni_{0.4}Cr_{0.6}O_3$ after calcined.

Figure S16. XRD patterns of 0.2Ce-$La_{0.97}Ni_{0.4}Cr_{0.6}O_3$.

Figure S17. AFM images of R-0.2Ce.

Figure S18. TEM images and EDS mapping of 0.4Ce-$La_{0.97}Ni_{0.4}Cr_{0.6}O_3$ after 10% H_2 reduction at 900°C for 3 h.

Figure S19. SEM images of 0.2Ce-$La_{0.97}Ni_{0.4}Cr_{0.6}O_3$ after reduced at 700 °C for 3 h.

Figure S20. SEM images of 0.2Ce-$La_{0.97}Ni_{0.4}Cr_{0.6}O_3$ after reduced at 800 °C for 3 h.

Figure S21. SEM images of 0.2Ce-$La_{0.97}Ni_{0.4}Cr_{0.6}O_3$ after reduced at 900 °C for 3 h.

Figure S22. SEM images of 0.2Ce-$La_{0.97}Ni_{0.4}Cr_{0.6}O_3$ after reduced at 1000 °C for 3 h.

Figure S23. Particle size of R-0.2Ce sample with different reduction temperatures.

Figure S24. Particle size and density distribution of R-0.2Ce sample with different reduction temperatures.

Figure S25. XRD pattern of R-0.2Ce sample with different reduction temperatures (Local magnification corresponds to the main perovskite peak, CeO_{2-x} and Ni^0 peaks).

Figure S26. The conversion rates of CH_4 and CO_2, selectivity of H_2 and CO, and H_2/CO value of sample R-0.2Ce at different reduction temperature.

Figure S27. AC-HAADF-STEM-EDS mapping of **a** La/Ni and **b** La/Cr elements.

Figure S28. XRD refinement data of calcined C-0.2Ce sample.

Figure S29. XRD refinement data of calcined C-0Ce sample.

Figure S30. XRD pattern of R-0.2Ce and R-0Ce samples.

Figure S31. Fourier transform of the k^3-weighted EXAFS curves (empty dots) and fit model (red line) for the reduced $La_{0.97}Ni_{0.4}Cr_{0.6}O_3$ samples. The light grey shadow indicates the fitted region.

Figure S32. Ni K-edge XANES spectra of 0.2Ce-$La_{0.97}Ni_{0.4}Cr_{0.6}O_3$ materials after exsolution with their linear combination fittings (LCF) containing the weighted components of the $LaNi_{0.4}Cr_{0.6}O_3$ after reduction, metallic Ni, the linear combination fitting and difference plots (residual) obtained from A_{exp}-A_{fit}, with A the normalized absorbance for the exsolved materials.

Figure S33. k^3-weighted wavelet transform plots of Ni K-edge EXAFS spectra of Ni-foil.

Figure S34. k^3-weighted wavelet transform plots of Ni K-edge EXAFS spectra of NiO.

Figure S35. Ni K-edge EXAFS (circle) and the fit curve (line) for **a** $LaNi_{0.4}Cr_{0.6}O_3$, **b** 0.2Ce-$La_{0.97}Ni_{0.4}Cr_{0.6}O_3$, **c** NiO, **d** Ni-foil shown in k^3-weighted k-space.

Figure S36. XAFS results of Ni K-edge EXAFS spectra ($\chi(k)k^3$) of $LaNi_{0.4}Cr_{0.6}O_3$, 0.2Ce-$La_{0.97}Ni_{0.4}Cr_{0.6}O_3$, NiO, and Ni-foil.

Figure S37. Cr 2*p* XPS spectra of the reduced *x*Ce-$La_{0.97}Ni_{0.4}Cr_{0.6}O_3$ perovskite.

Figure S38. Raman spectra of the reduced *x*Ce-$La_{0.97}Ni_{0.4}Cr_{0.6}O_3$ perovskite.

Figure S39. **a** O 1*s*, **b** Ni 3*p*, **c** Cr 2*p* XPS spectra of fresh $LaNi_{0.4}Cr_{0.6}O_3$, after CH_4 treatment, and after CO_2 treatment.

Figure S40. **a** O 1*s*, **b** Ce 3*d*, **c** Ni 3*p*, **d** Cr 2*p* XPS spectra of fresh 0.2Ce-$La_{0.97}Ni_{0.4}Cr_{0.6}O_3$, after CH_4 treatment, and after CO_2 treatment.

Figure S41. **a** O 1*s*, **b** Ce 3*d*, **c** Ni 3*p*, **d** Cr 2*p* XPS spectra of fresh 0.4Ce-$La_{0.97}Ni_{0.4}Cr_{0.6}O_3$, after CH_4 treatment, and after CO_2 treatment.

Figure S42. MS signal of CH_4/CO_2 alternately pulse experiments at 750 °C on 0.4Ce-$La_{0.97}Ni_{0.4}Cr_{0.6}O_3$.

Figure S43. CH_4-TPSR of 0.4Ce-$La_{0.97}Ni_{0.4}Cr_{0.6}O_3$.

Figure S44. XRD pattern of fresh and 120h-reacted R-0Ce.

Figure S45. Raman spectra of 120h-reacted R-0Ce and 800h-reacted R-0.2Ce.

Figure S46. **a** SEM images of 800h-reacted R-0.2Ce. **b** Particle size of R-0.2Ce. **c** SEM images of 120h-reacted R-0 Ce. **d** Particle size of R-0Ce.

Figure S47. TEM images and EDS mapping of 800h-reacted R-0.2Ce.

Figure S48. In-situ DRIFTS results of 0.4Ce-$La_{0.97}Ni_{0.4}Cr_{0.6}O_3$.

Figure S49. The structures of initial states and final states for CH_2 dissociation into CH^* or reacting with O to CH_2O^* on the R-0Ce and R-0.2Ce catalysts.

Figure S50. Surface morphology of R-0Ce and R-0.2Ce catalysts. Red, light blue, blue-gray, steel blue, and beige balls represent O, La, Cr, Ni, and Ce atoms, respectively.

Table S1. Comparison of the DRM performance with literature.

Table S2. The ICP-OES results of fresh samples.

Table S3. The XRD Rietveld refinement results, unit cell parameters and oxygen deficiency of 0.2Ce sample.

Table S4. The XRD Rietveld refinement results, unit cell parameters and oxygen deficiency of 0Ce sample.

Table S5. Results of the fitting of the EXAFS curves for Ni K-edge EXAFS. The experimental data (k-range: 2.5-14.0 $Å^{-1}$, R-range: 1.0-3.3 Å) were fitted in R-space (k-weighting=3).

Table S6. Summary of the linear combination fittings (LCF) from the XANES results

Table S7. Summary of XPS Characteristics of reduced samples.

Table S8. Summary of CH_4, CO_2 treated XPS Characteristics.

Table S9. Bader charge of Ni atoms (*e*), as well as the total Bader charge of Ni_4 cluster on the R-0Ce and R-0.2Ce catalysts.

Table S10. Bader charge of Ce atoms (*e*), as well as the total Bader charge of CeO_{2-x} cluster on the R-0Ce and R-0.2Ce catalysts.

Table S11. Detailed list of abbreviations.

1. DRM performance of R-*x*Ce

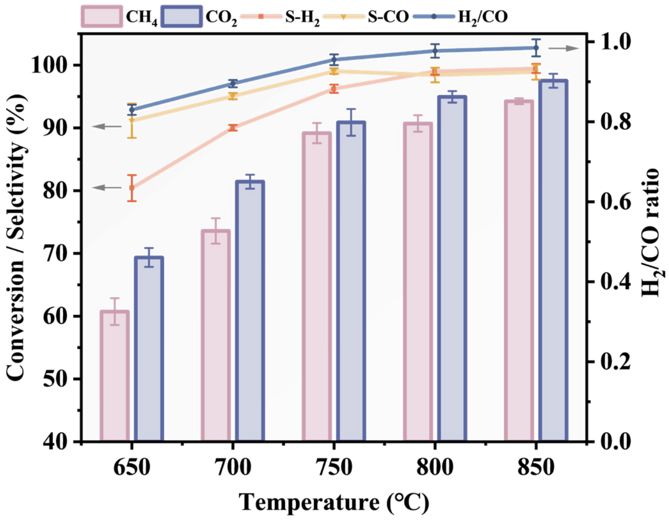

Figure S1. The conversion rates of CH_4 and CO_2, selectivity of H_2 and CO, and H_2/CO ratio at different temperatures for sample R-0.2Ce at WHSV=24000 mL·g^{-1}·h^{-1}.

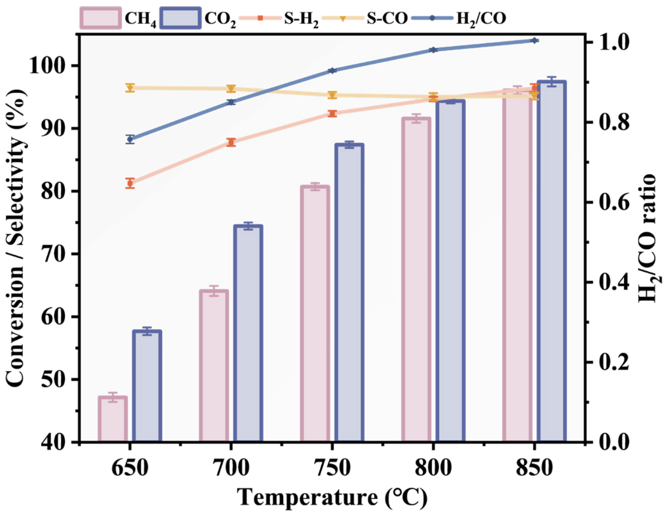

Figure S2. The conversion rates of CH_4 and CO_2, selectivity of H_2 and CO, and H_2/CO ratio at different temperatures for sample R-0Ce at WHSV=12000 $mL\cdot g^{-1}\cdot h^{-1}$.

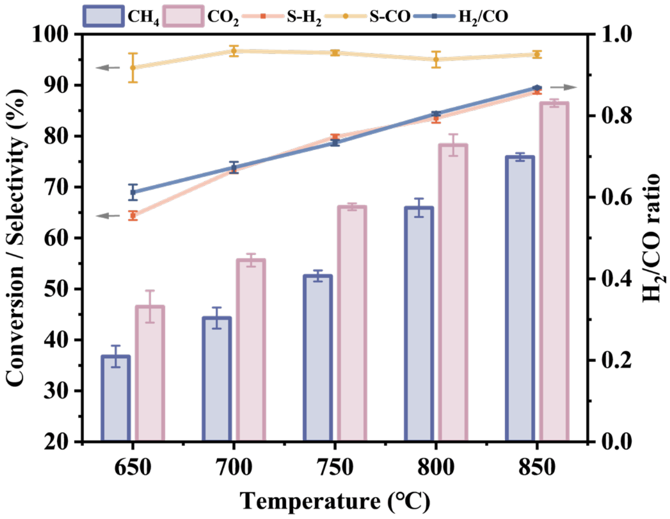

Figure S3. The conversion rates of CH_4 and CO_2, selectivity of H_2 and CO, and H_2/CO ratio at different temperatures for sample R-0.4Ce at WHSV=12000 $mL\cdot g^{-1}\cdot h^{-1}$.

As a control group, we also tested the catalytic activity of R-0Ce and R-0.4Ce samples at different temperatures, as shown in Fig. S2, Fig. S3. R-0Ce samples still have good activity at high temperatures, but its CO selectivity is relatively low, and there may be a side reaction of CO disproportionation and CH_4 decomposition of carbon accumulation occurring. The catalytic activity of the R-0.4Ce sample at all temperatures has a large gap compared with the other samples, which implies that it has a significant effect of Ce doping ratio on the performance of the exsolved perovskite catalysts.

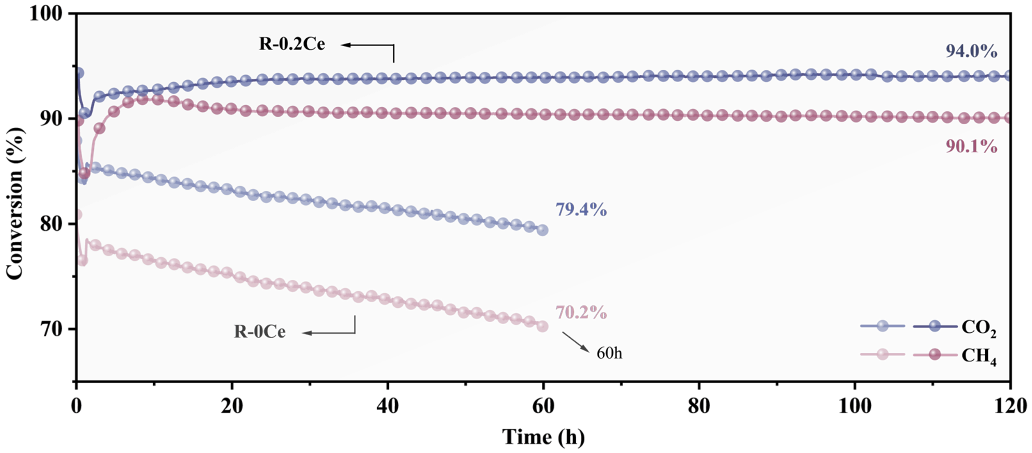

Figure S4. CH_4 and CO_2 conversion rate of stability test of R-0.2Ce and R-0Ce for 120 h and 60 h. Evaluated conditions: 750 °C, CH_4: CO_2: N_2=1: 1:2, WHSV=12000 mL·g^{-1}·h^{-1}.

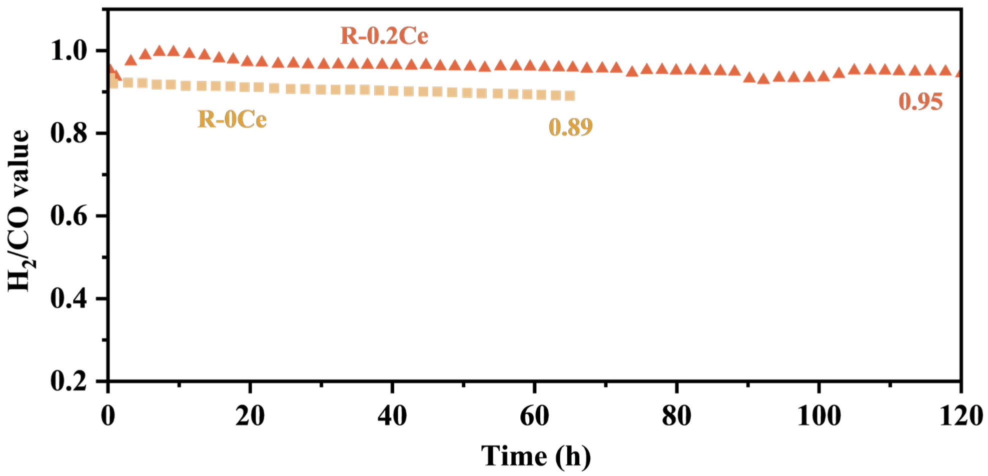

Figure S5. H_2/CO value of stability test of R-0.2Ce and R-0Ce for 120 h and 60 h. Evaluated conditions: 750 ℃, CH_4: CO_2: N_2=1: 1: 2, WHSV=12000 mL·g^{-1}·h^{-1}.

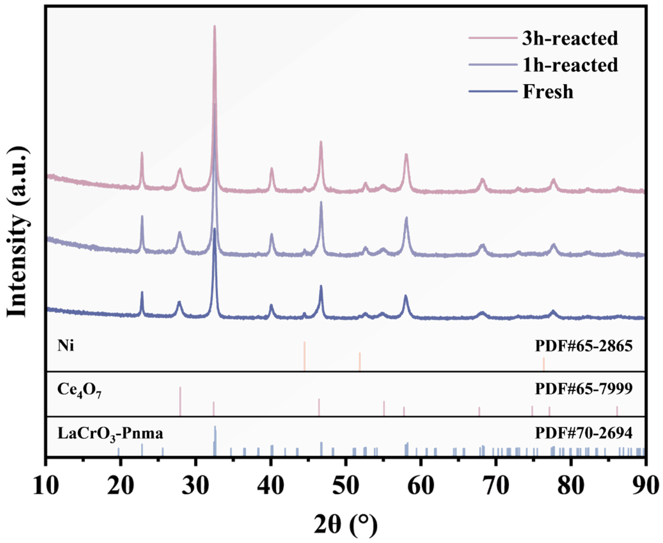

Figure S6. XRD patterns of fresh, 1h DRM reacted, and 3h DRM reacted 0.2Ce-$La_{0.97}Ni_{0.4}Cr_{0.6}O_3$.

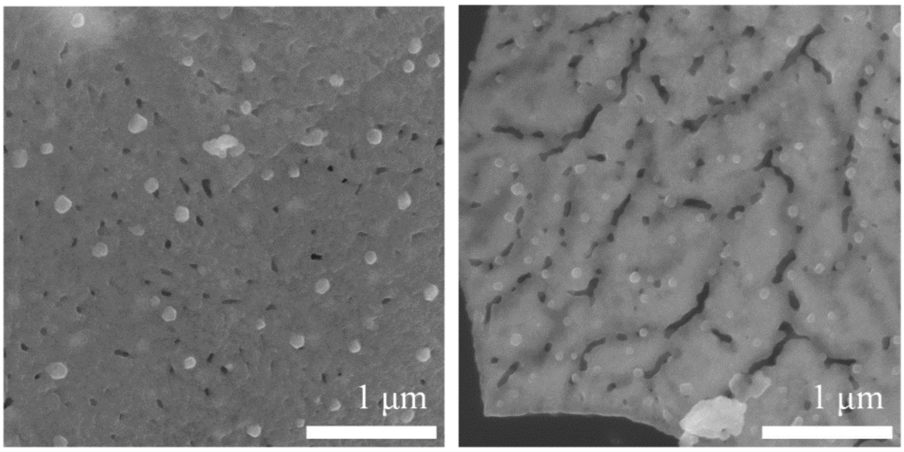

Figure S7. SEM images of fresh $0.2Ce\text{-}La_{0.97}Ni_{0.4}Cr_{0.6}O_3$.

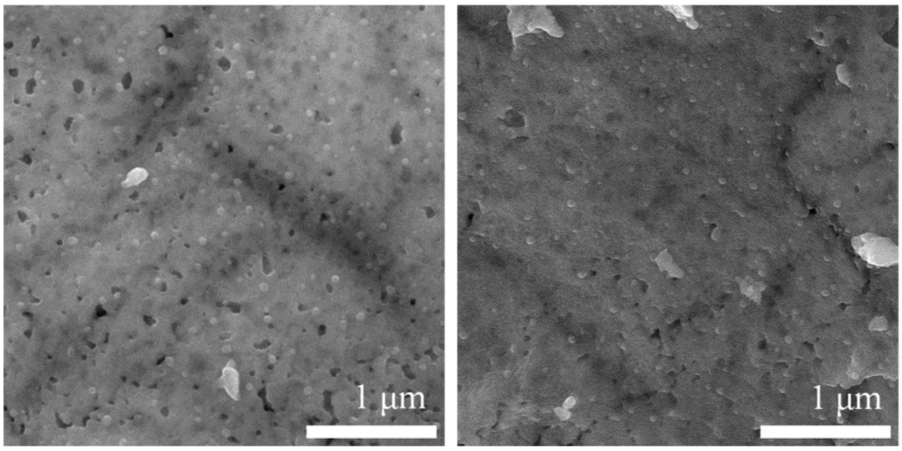

Figure S8. SEM images of 1 h DRM reacted 0.2Ce-$La_{0.97}Ni_{0.4}Cr_{0.6}O_3$.

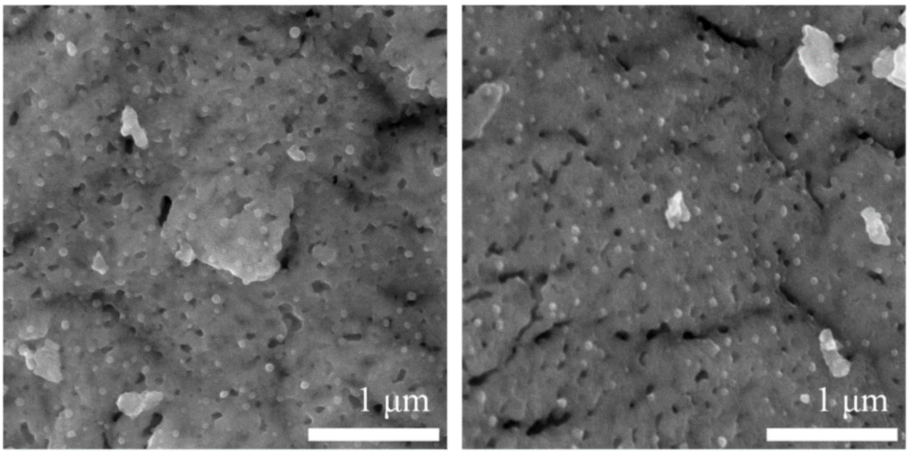

Figure S9. SEM images of 3 h DRM reacted $0.2Ce\text{-}La_{0.97}Ni_{0.4}Cr_{0.6}O_3$.

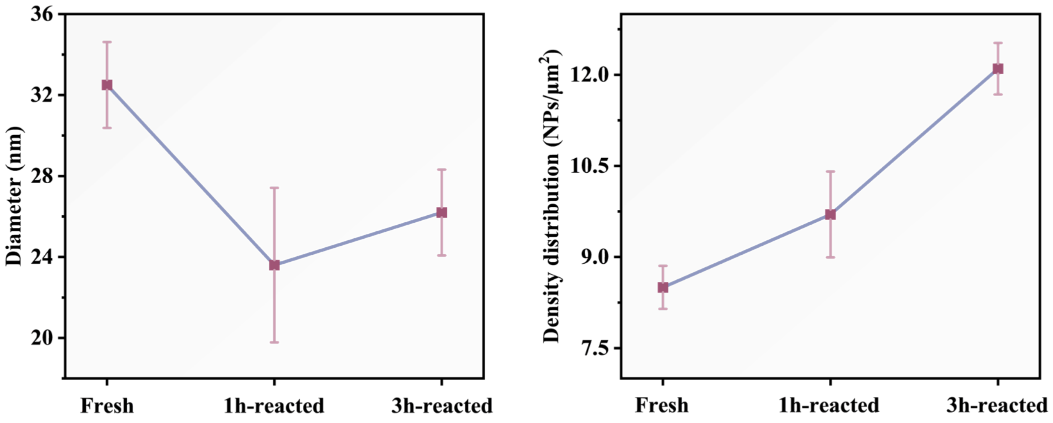

Figure S10. Particle size and density distribution of R-0.2Ce sample with different DRM reaction time (Obtained by ImageJ software through SEM image statistics, the standard deviation error bars were obtained by statistically analyzing the nanoparticle size and density distributions from three SEM images taken from different regions of each sample).

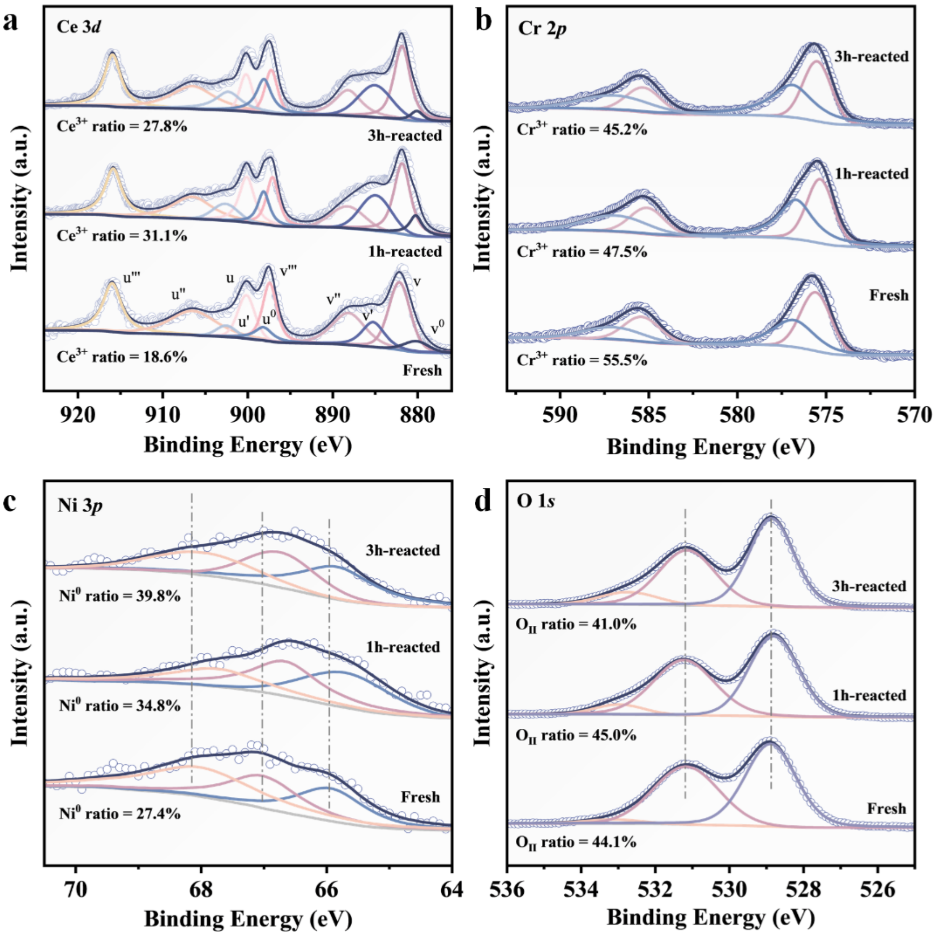

Figure S11. a Ce 3*d*, **b** Cr 2*p*, **c** Ni 3*p*, **d** O 1*s* XPS spectra of fresh 0.2Ce-$La_{0.97}Ni_{0.4}Cr_{0.6}O_3$, after 1 h DRM reacted, and after 3 h DRM reacted.

It is worth noting that there is a considerable variation in the DRM performance of the samples during the initial 3 h. In conjunction with XRD, SEM and XPS characterization (Figs S6-S11), it is presumed that this is attributable to the decline in the strength of the perovskite B-O bond in the early stage of the reaction. It is due to the local destruction of the bond (an increase in the proportion of O_{ads}), which affects the structural stability of materials, and by extension, the catalytic performance. Simultaneously, this also decreases the energy barrier for the Ni exsolution, which leads to a significant enhancement of the density distribution of the Ni nanoparticles in the

samples after the 3h reaction. Concurrently, the Ni-O bond strength increased and the O_{ads} slightly decreased, indicating that the oxygen vacancies in the interface and the matrix were filled. Ni 3*p* spectra shift toward higher binding energy suggesting the electron exchange at the interface between Ni^0 and the matrix was enhanced. It forms a more stable interfacial bonding state. Then the catalytic performance was restored and maintained stable for a long period of time.

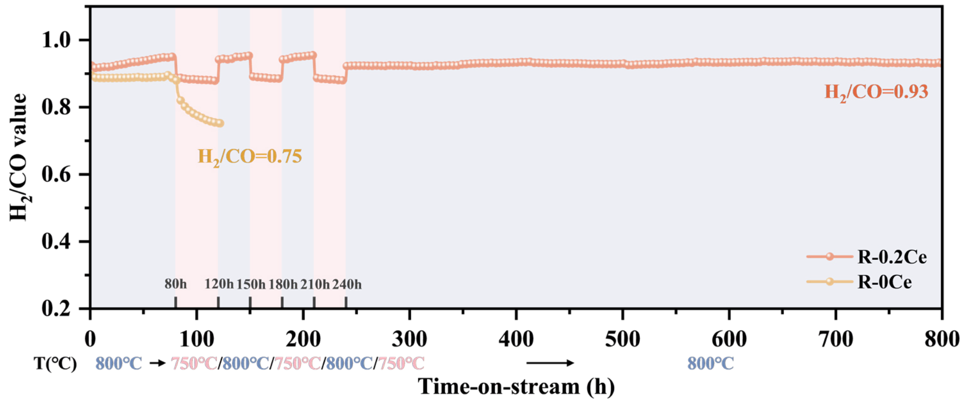

Figure S12. The H_2/CO value of R-0.2Ce and R-0Ce in the DRM stability test.

It can be seen (Fig. S12) that the H_2/CO ratio of R-0.2Ce stays around 0.93 (800 °C) after 800 h, and the H_2/CO ratio is 0.88 (750 °C) at 240 h. For comparison, the H_2/CO value of the R-0Ce after 120 h is only 0.75 (750 °C). This result indicate that Ce modification hinders the side reactions of DRM process.

2. Microstructure, morphology, and phase analysis of R-*x*Ce

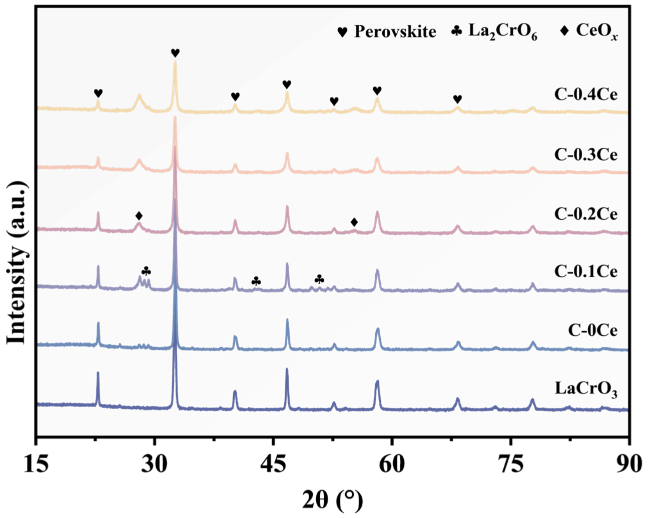

Figure S13. XRD patterns of calcined $LaCrO_3$, $LaNi_{0.4}Cr_{0.6}O_3$ and xCe-$La_{0.97}Ni_{0.4}Cr_{0.6}O_3$.

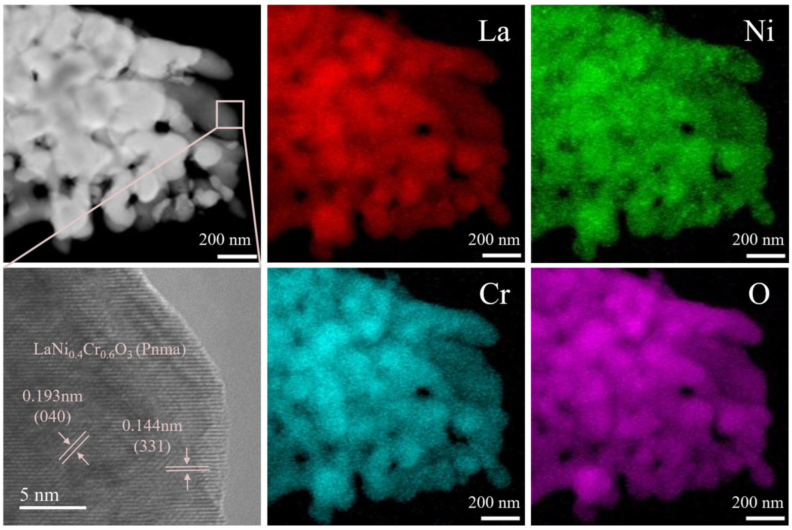

Figure S14. TEM images and EDX mapping of $LaNi_{0.4}Cr_{0.6}O_3$ after 800 °C, 4 h calcination.

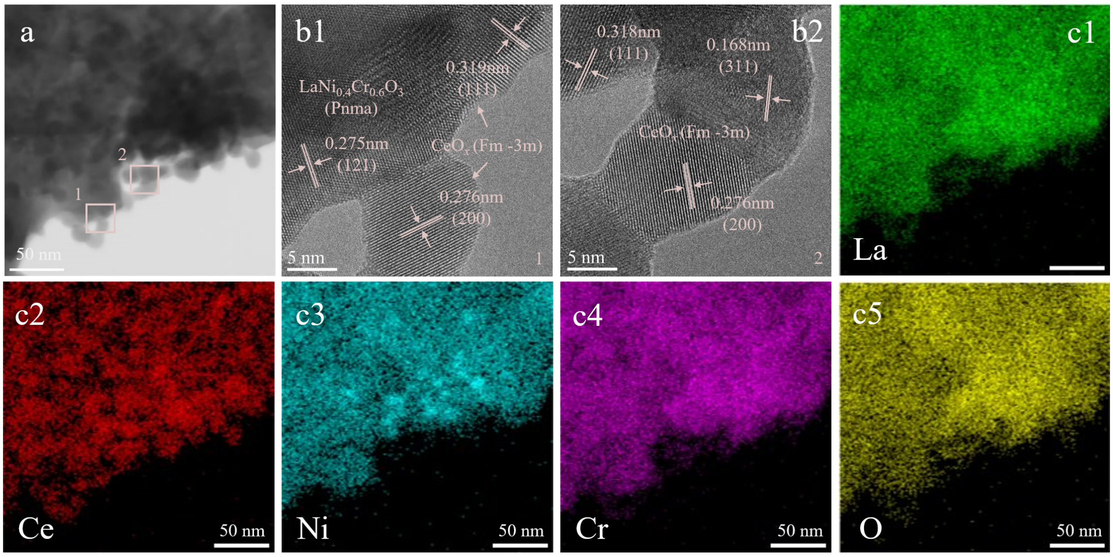

Figure S15. TEM images and EDX mapping of 0.2Ce-$La_{0.97}Ni_{0.4}Cr_{0.6}O_3$ after calcination.

The calcined samples are shown in Figs. S14-S15, with a uniform distribution of lanthanum, nickel, chromium, and oxygen. The perovskite phase primarily exhibits the (121) facet, and CeO_{2-x} mainly exposes the (200) and (111) facets. When considered alongside the XRD results of sample 0.2Ce, it can be seen that some of the cerium covered the surface of the perovskite in the form of CeO_{2-x}, and a bridge-like structure was formed.

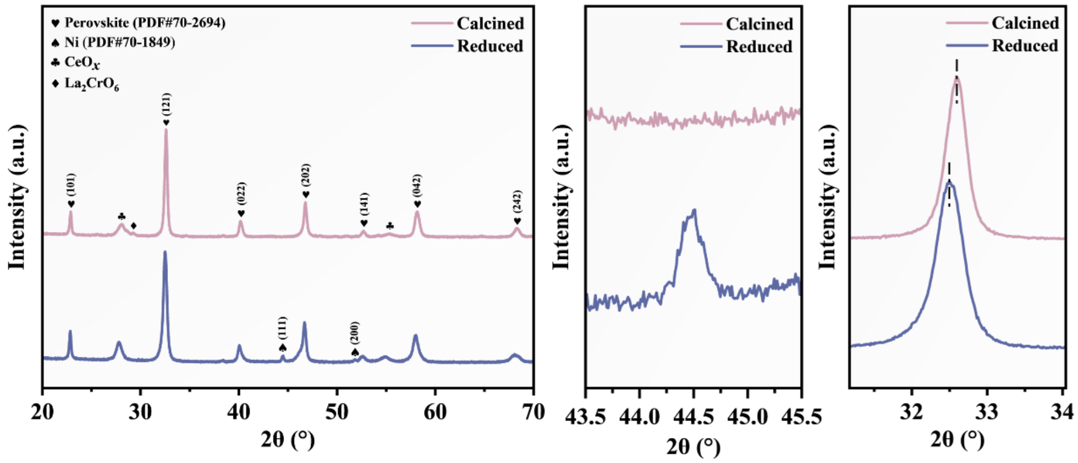

Figure S16. XRD patterns of 0.2Ce-$La_{0.97}Ni_{0.4}Cr_{0.6}O_3$.

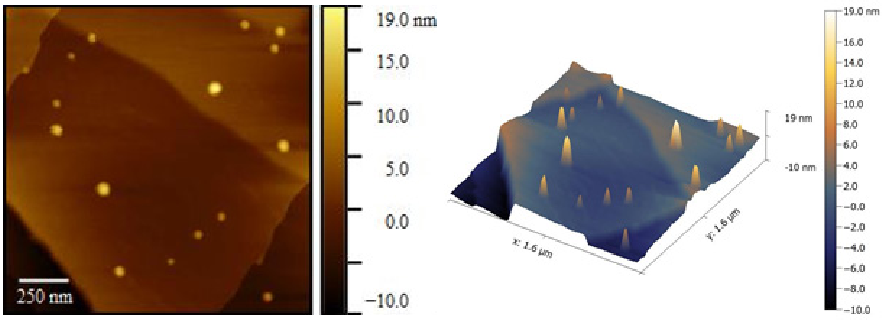

Figure S17. AFM images of R-0.2Ce.

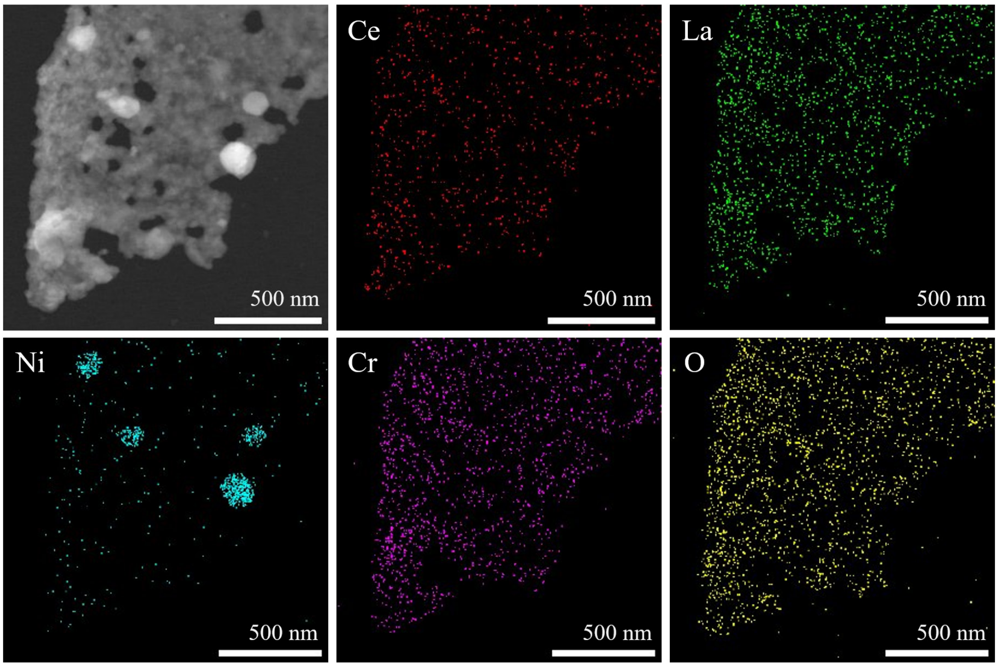

Figure S18. TEM images and EDX mapping of 0.4Ce-$La_{0.97}Ni_{0.4}Cr_{0.6}O_3$ after 10% H_2 reduction at 900 °C for 3 h.

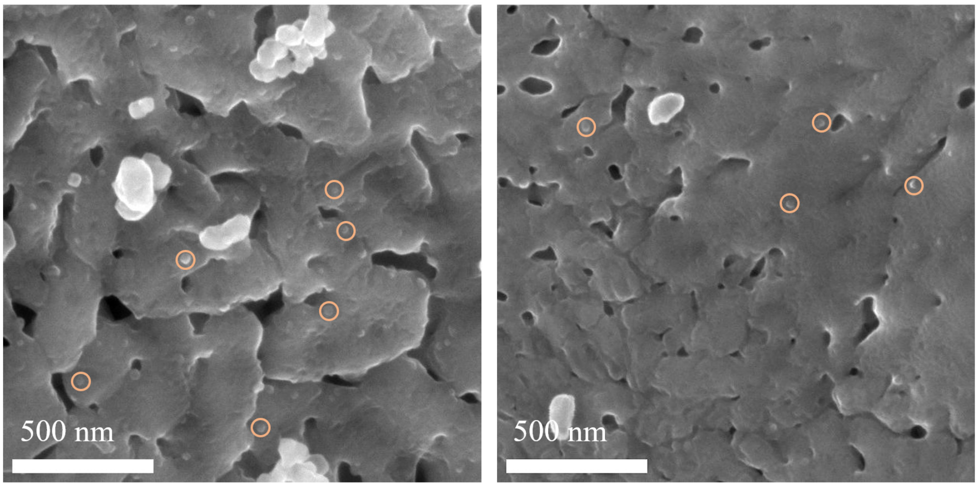

Figure S19. SEM images of $0.2Ce\text{-}La_{0.97}Ni_{0.4}Cr_{0.6}O_3$ after reduction at 700 ℃ for 3 h (The highlighted circles are exsolved nanoparticles).

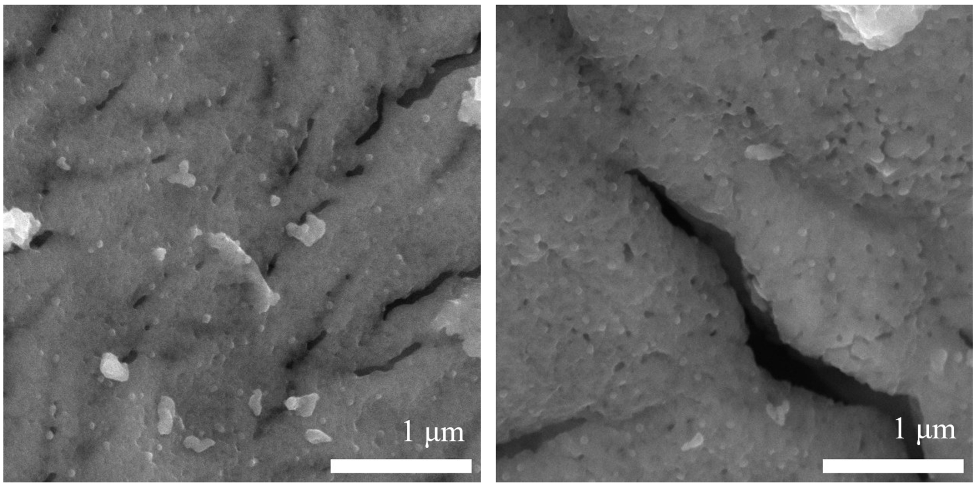

Figure S20. SEM images of 0.2Ce-$La_{0.97}Ni_{0.4}Cr_{0.6}O_3$ after reduction at 800 °C for 3 h.

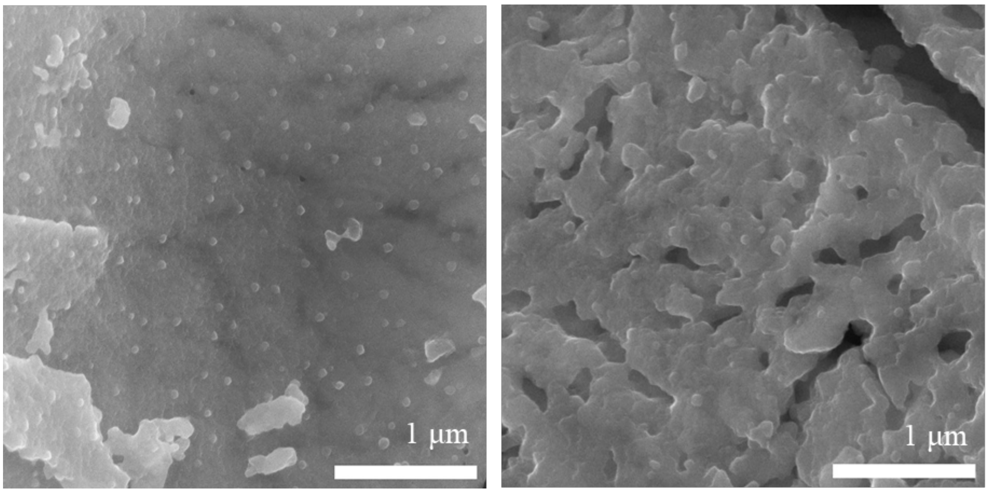

Figure S21. SEM images of 0.2Ce-$La_{0.97}Ni_{0.4}Cr_{0.6}O_3$ after reduction at 900 ℃ for 3 h.

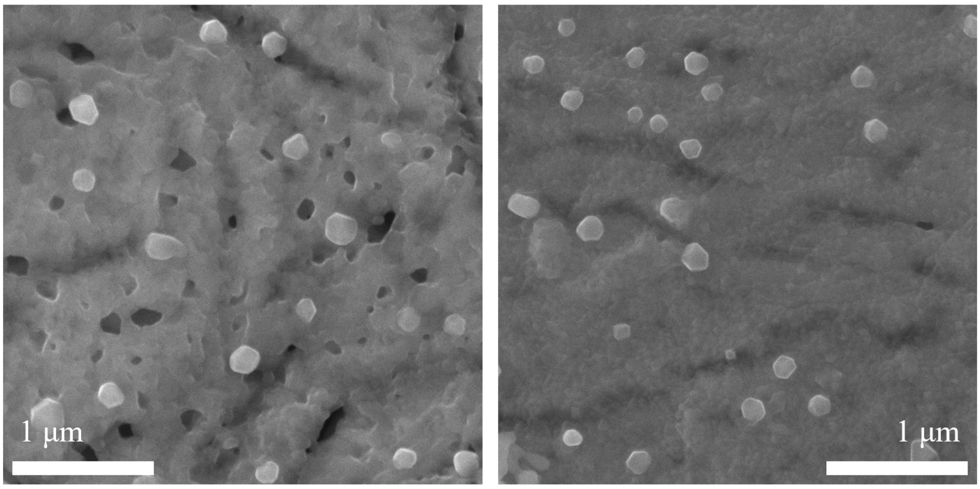

Figure S22. SEM images of 0.2Ce-$La_{0.97}Ni_{0.4}Cr_{0.6}O_3$ after reduction at 1000 °C for 3 h.

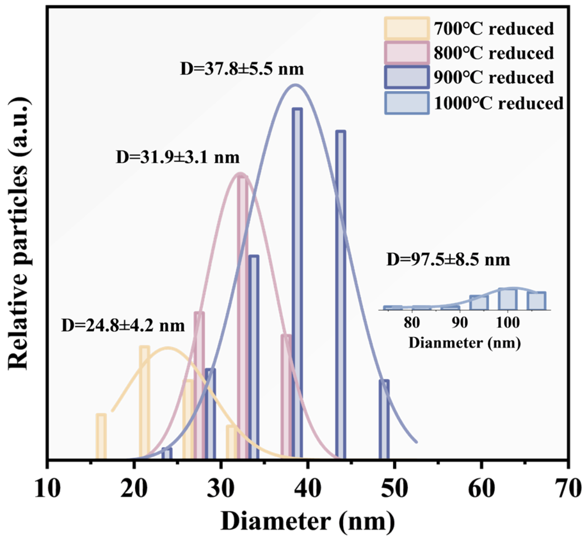

Figure S23. Particle size of R-0.2Ce sample with different reduction temperatures.

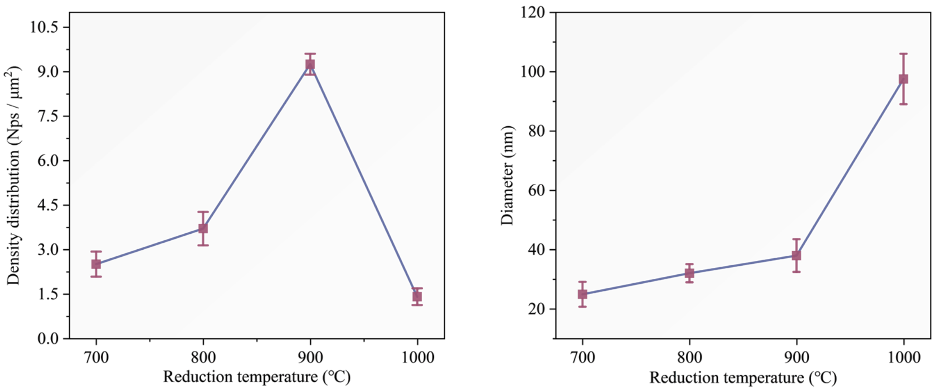

Figure S24. Particle size and density distribution of R-0.2Ce sample with different reduction temperatures (the standard deviation error bars were obtained by statistically analyzing the nanoparticle size and density distributions from three SEM images taken from different regions of each sample).

The density of exsolved nanoparticles demonstrates a gradual increase with rising reduction temperature, reaching a maximum value of approximately 9.3 NPs/μm^2 at 900 °C. Thereafter, upon further elevation of the reduction temperature to 1000 °C, the density distribution exhibits a pronounced decline, approaching the level observed in the reduced sample at 700 °C (≈2.2 NPs/μm^2). The alteration in particle size is relatively minor, increasing slightly from 700 °C (24.8 nm) to 900 °C (37.98 nm). The particle size of the exsolved nanoparticles in the 1000 °C reduced sample has increased almost threefold, reaching 97.5 nm. Additionally, the SEM image reveals the presence of large particles on the surface of the sample.

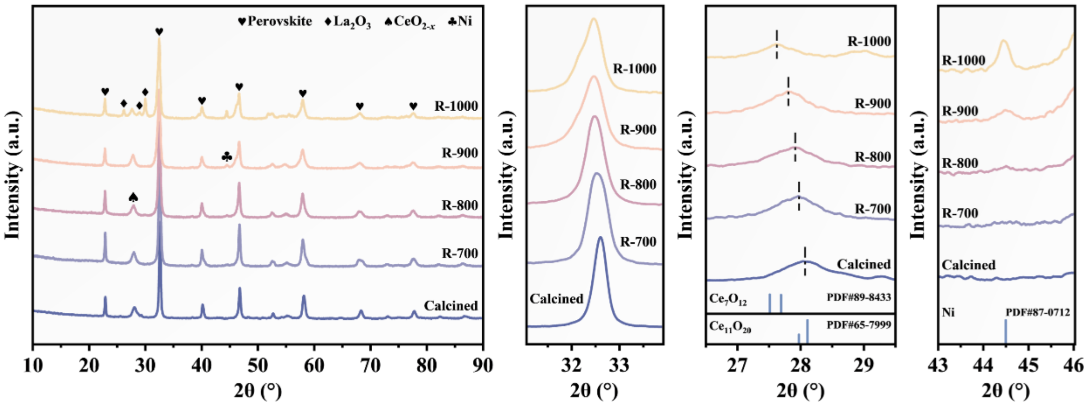

Figure S25. XRD pattern of R-0.2Ce sample with different reduction temperatures (Local magnification corresponds to the main perovskite peak, CeO_{2-x} and Ni^0 peaks).

A comparison of the calcined samples revealed that the main peak of perovskite shifted to a lower angle with increasing reduction temperature, and the Ni^0 peak at 44.5° gradually emerged. This indicated that the process of exsolution was deepening gradually, and that the Ni of small radius at the B-site was segregation from the perovskite bulk phase to surface. This resulted in an increase in the lattice constants. It is noteworthy that the main peak of perovskite has essentially no longer shift after 900 °C. It is postulated that the reduction at 900 °C has already reached the maximum amount of Ni exsolution within the system. Continued elevation of the reduction temperature is predicted to promote the growth of nanoparticles and lead to the partial decomposition of the perovskite phase, formed La_2O_3. Furthermore, the average oxidation state of Ce was observed to decrease with increasing reduction temperature. This was evidenced by a low-angle shift from the $Ce_{11}O_{20}$ standard card, which corresponded to the initial fresh sample, to the PDF standard card of Ce_7O_{12} in XRD.

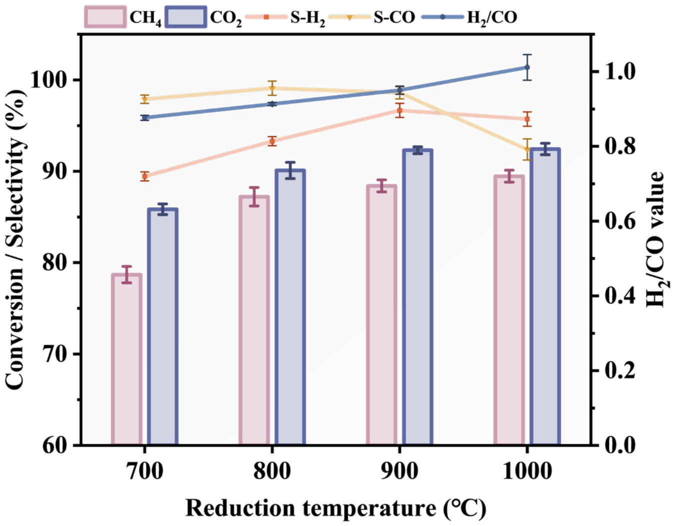

Figure S26. The conversion rates of CH_4 and CO_2, selectivity of H_2 and CO, and H_2/CO value of sample R-0.2Ce at different reduction temperature. (750 °C, CH_4: CO_2: N_2=1: 1: 2, WHSV=12000 mL·g^{-1}·h^{-1})

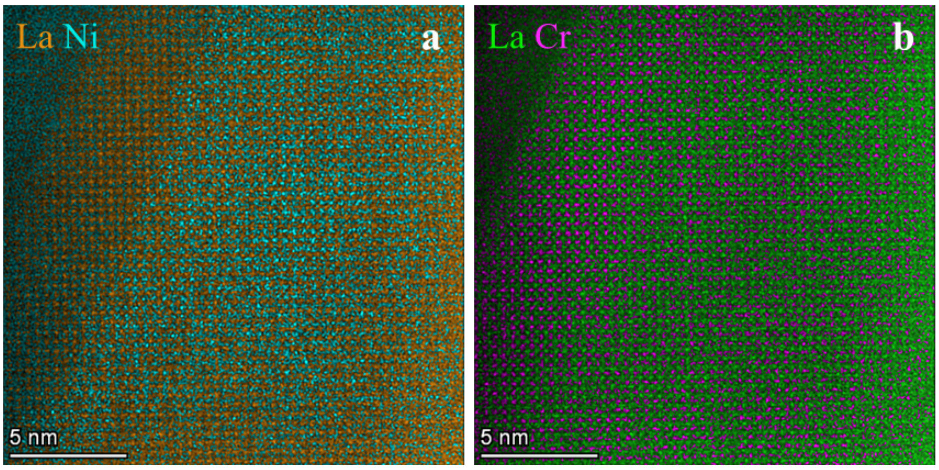

Figure S27. AC-HAADF-STEM-EDS mapping of **a** La/Ni and **b** La/Cr elements.

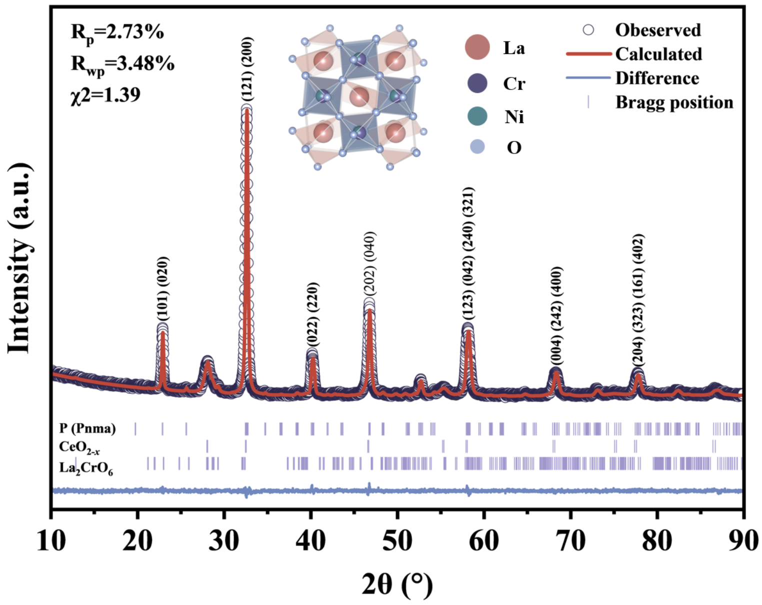

Figure S28. XRD refinement data of calcined C-0.2Ce sample.

The perovskite tolerance factor[1] is employed to indicate the extent of deviation from the ideal cubic structure (t=1):

$$t = \frac{(r_A + r_O)}{\sqrt{2}(r_B + r_O)} \quad (1)$$

where r_A, r_B, r_O represent the A-site, B-site, and O-ionic radii, respectively. When the tolerance factor is between 0.75 and 1, perovskite structures exhibiting orthorhombic or rhombohedral distortions are formed[2].

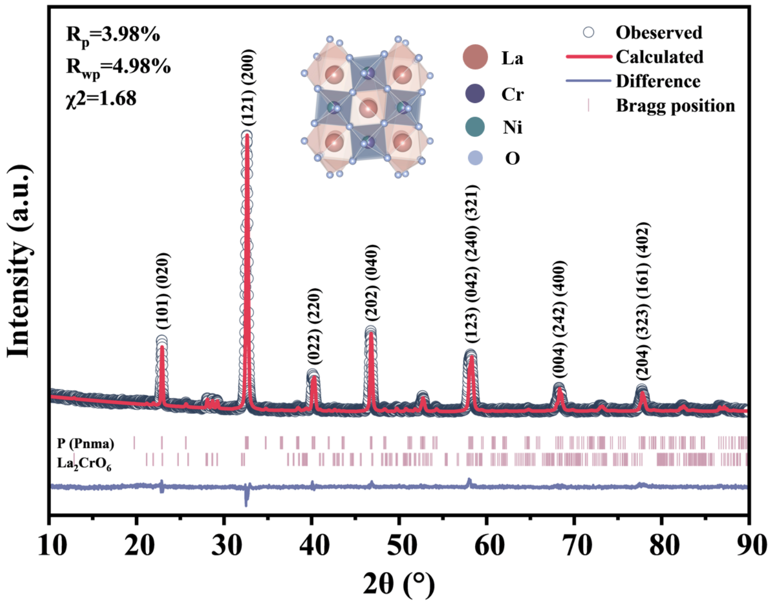

Figure S29. XRD refinement data of calcined C-0Ce sample.

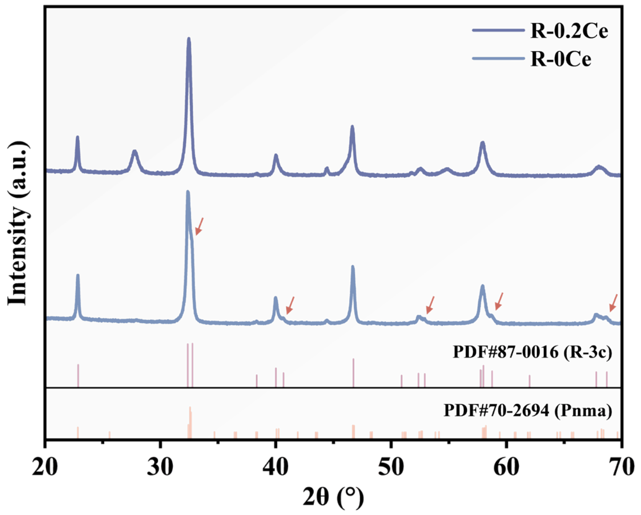

Figure S30. XRD pattern of R-0.2Ce and R-0Ce samples (The arrows indicate the characteristic peaks corresponding to R-3c space group).

3. Fine-structure characterizations of R-0Ce and R-0.2Ce

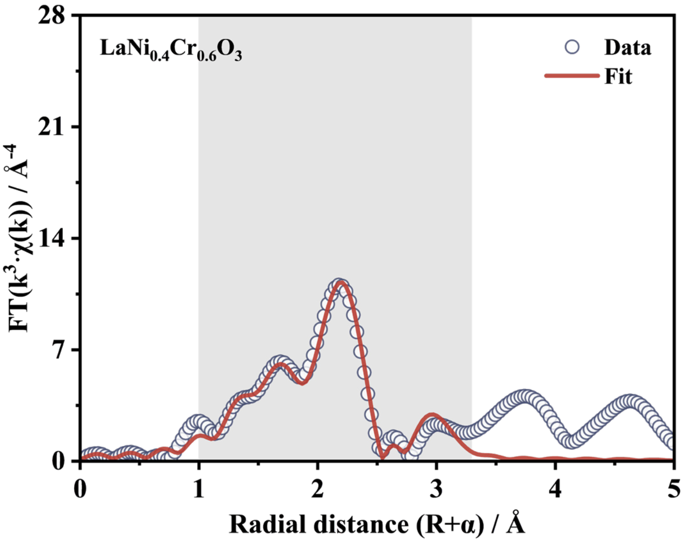

Figure S31. Fourier transform of the Ni k^3-weighted EXAFS curves (empty dots) and fit model (red line) for the reduced $LaNi_{0.4}Cr_{0.6}O_3$ samples. The light grey shadow indicates the fitted region.

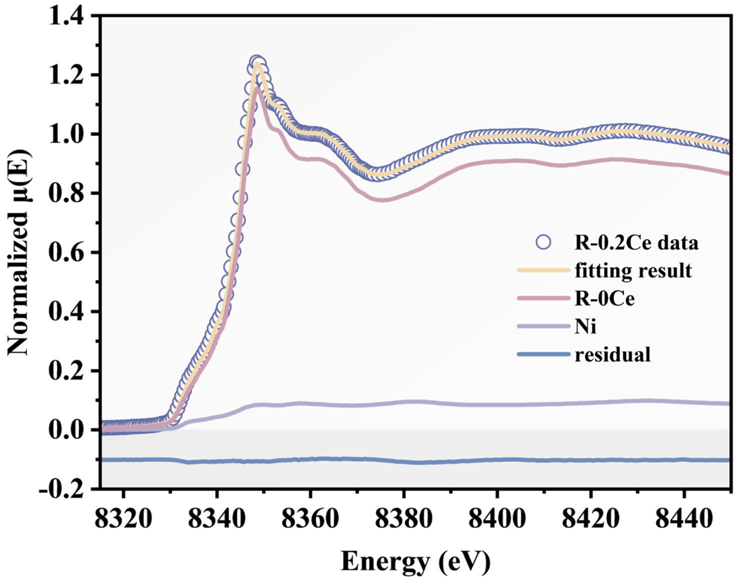

Figure S32. Ni K-edge XANES spectra of 0.2Ce-$La_{0.97}Ni_{0.4}Cr_{0.6}O_3$ materials after exsolution with their linear combination fittings (LCF) containing the weighted components of the $LaNi_{0.4}Cr_{0.6}O_3$ after reduction, metallic Ni, the linear combination fitting and difference plots (residual) obtained from A_{exp}-A_{fit}, with A the normalized absorbance for the exsolved materials.

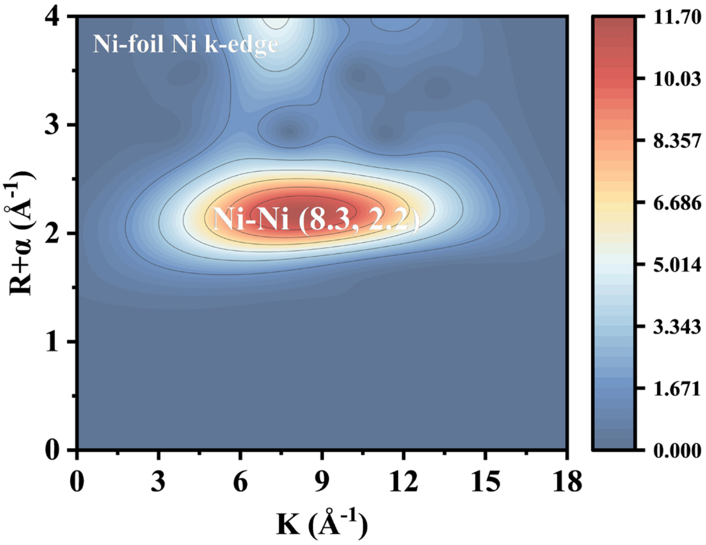

Figure S33. k^3-weighted wavelet transform plots of Ni K-edge EXAFS spectra of Ni-foil.

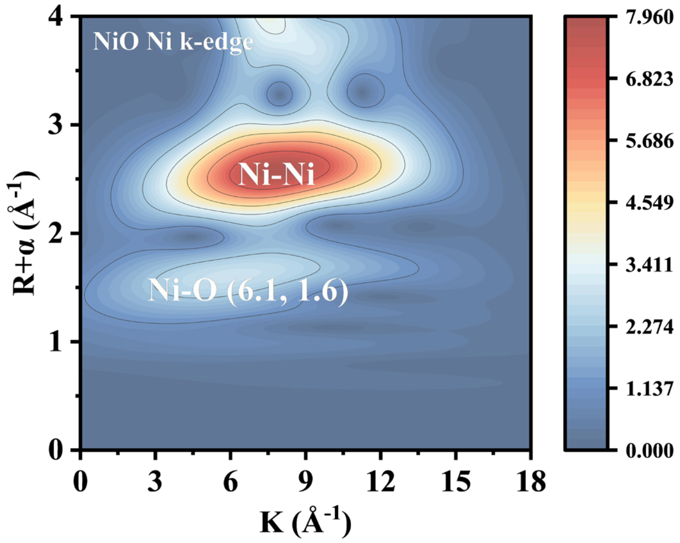

Figure S34. k^3-weighted wavelet transform plots of Ni K-edge EXAFS spectra of NiO.

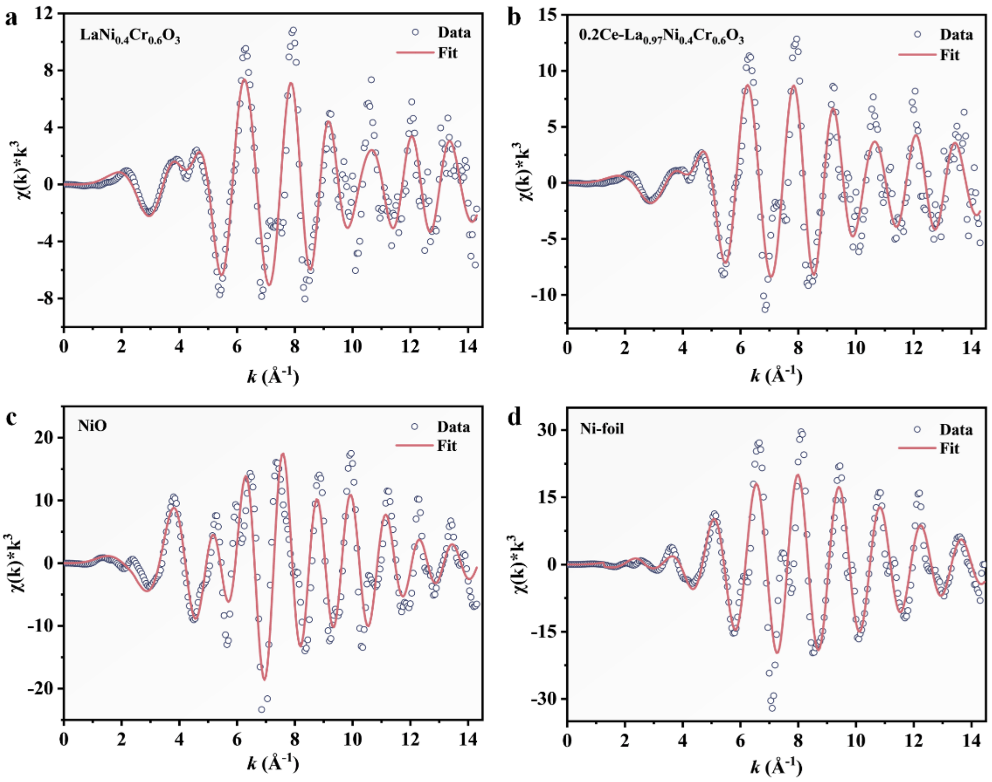

Figure S35. Ni K-edge EXAFS (circle) and the fit curve (line) for **a** $La_{0.97}Ni_{0.4}Cr_{0.6}O_3$, **b** 0.2Ce-$La_{0.97}Ni_{0.4}Cr_{0.6}O_3$, **c** NiO, **d** Ni-foil shown in k^3-weighted k-space.

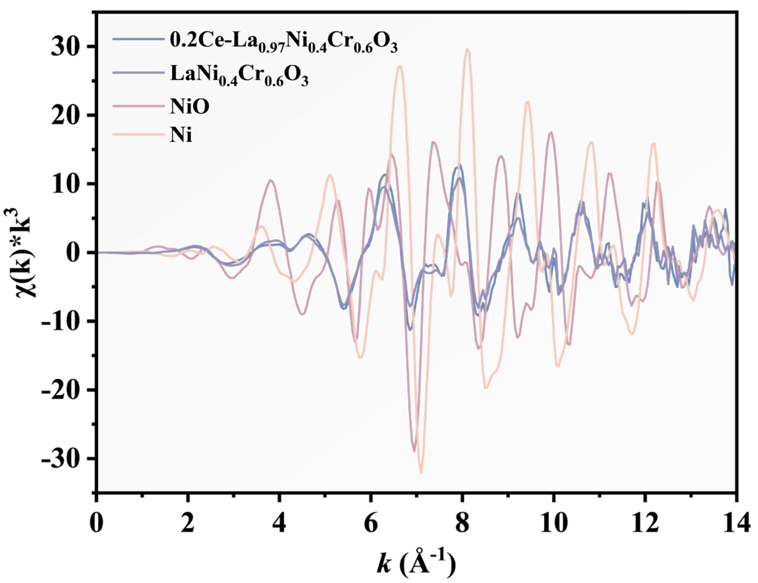

Figure S36. XAFS results of Ni K-edge EXAFS spectra ($\chi(k)k^3$) of $La_{0.97}Ni_{0.4}Cr_{0.6}O_3$, 0.2Ce-$La_{0.97}Ni_{0.4}Cr_{0.6}O_3$, NiO, and Ni-foil.

4. Effect of Ce-doping on structure of R-*x*Ce

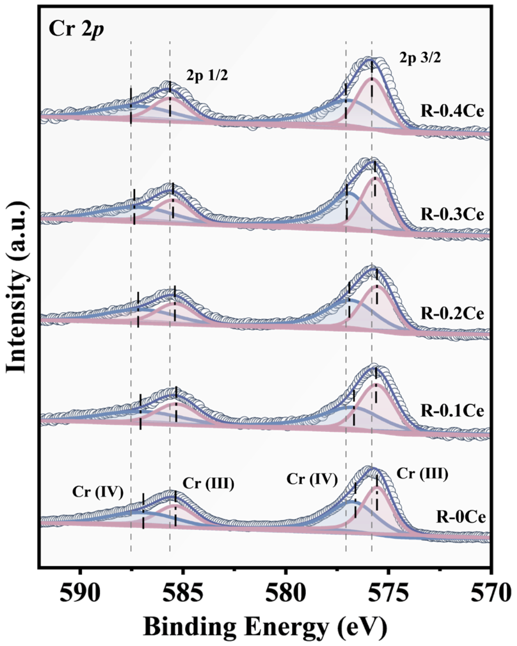

Figure S37. Cr 2*p* XPS spectra of the reduced *x*Ce-$La_{0.97}Ni_{0.4}Cr_{0.6}O_3$ perovskite.

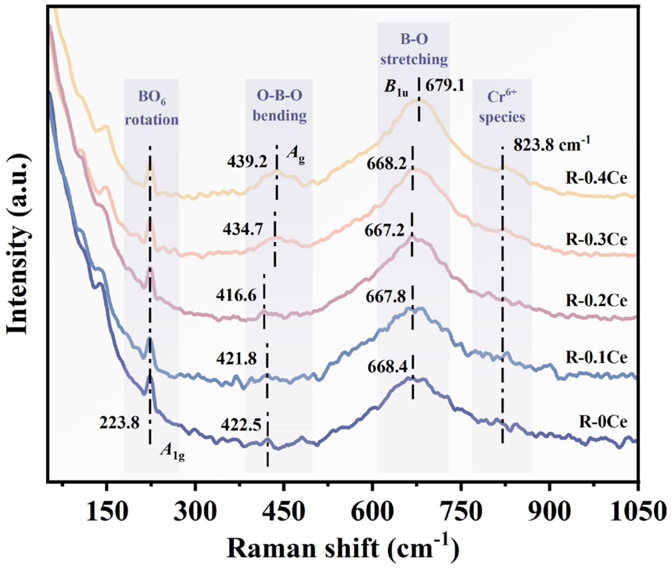

Figure S38. Raman spectra of the reduced *x*Ce-$La_{0.97}Ni_{0.4}Cr_{0.6}O_3$ perovskite.

The peaks in the Raman spectra associated with B-O-B stretching remain essentially unchanged for R-0Ce, R-0.1Ce, and R-0.2 Ce, then had a slightly blueshift under higher Ce doping. In contrast, the peaks corresponding to B-O-B bending exhibit a slight redshift, followed by a significant blueshift. This suggests that the average strength of the B-O bond undergoes a process of first decreasing and then increasing[3]. This trend is consistent with the shifting pattern of the binding energies of Ni 3*p*, and Cr 2*p*, and O 1*s* derived from XPS.

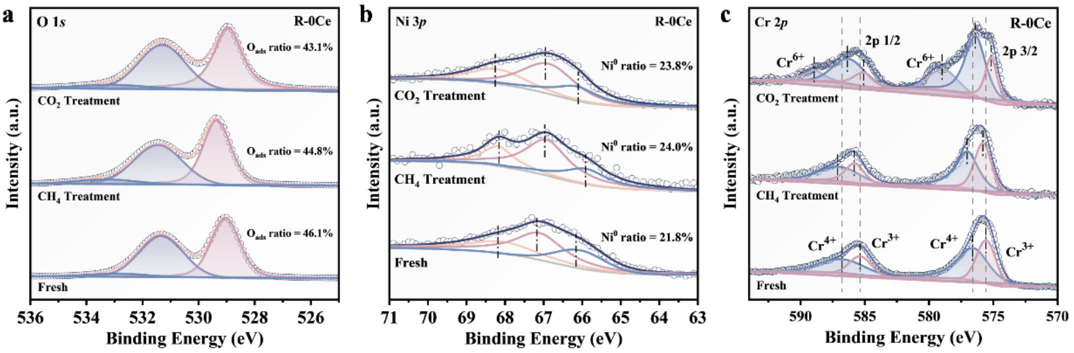

Figure S39. **a** O 1*s*, **b** Ni 3*p*, **c** Cr 2*p* XPS spectra of fresh $LaNi_{0.4}Cr_{0.6}O_3$, after CH_4 treatment, and after CO_2 treatment.

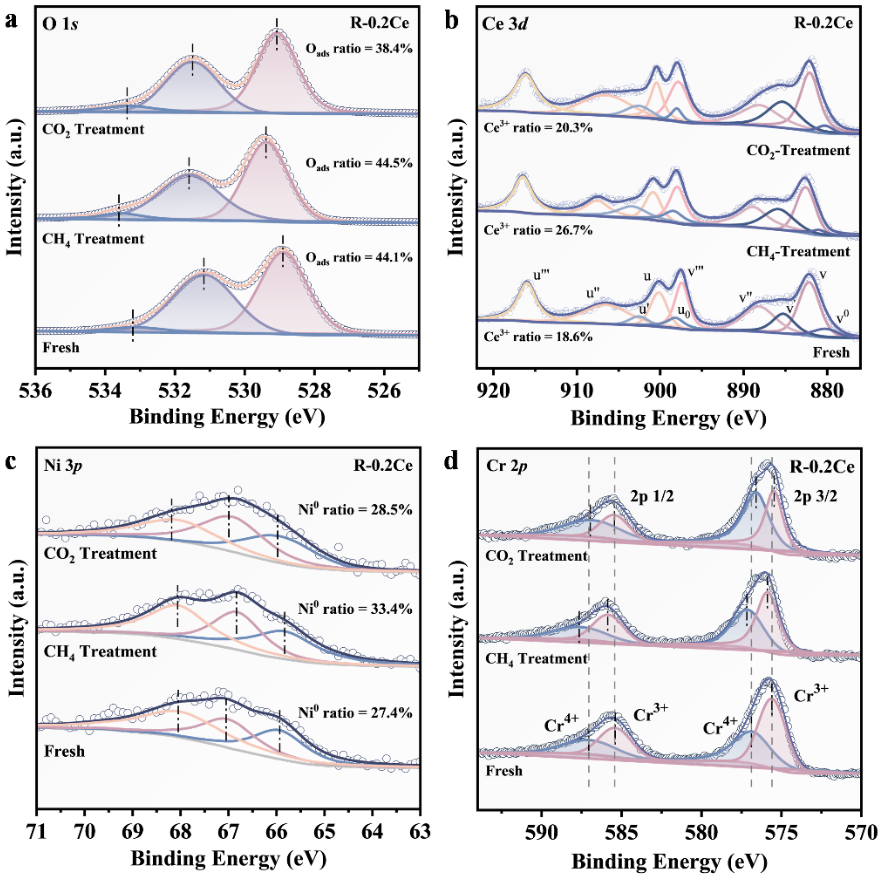

Figure S40. a O 1*s*, **b** Ce 3*d*, **c** Ni 3*p*, **d** Cr 2*p* XPS spectra of fresh 0.2Ce-$La_{0.97}Ni_{0.4}Cr_{0.6}O_3$, after CH_4 treatment, and after CO_2 treatment.

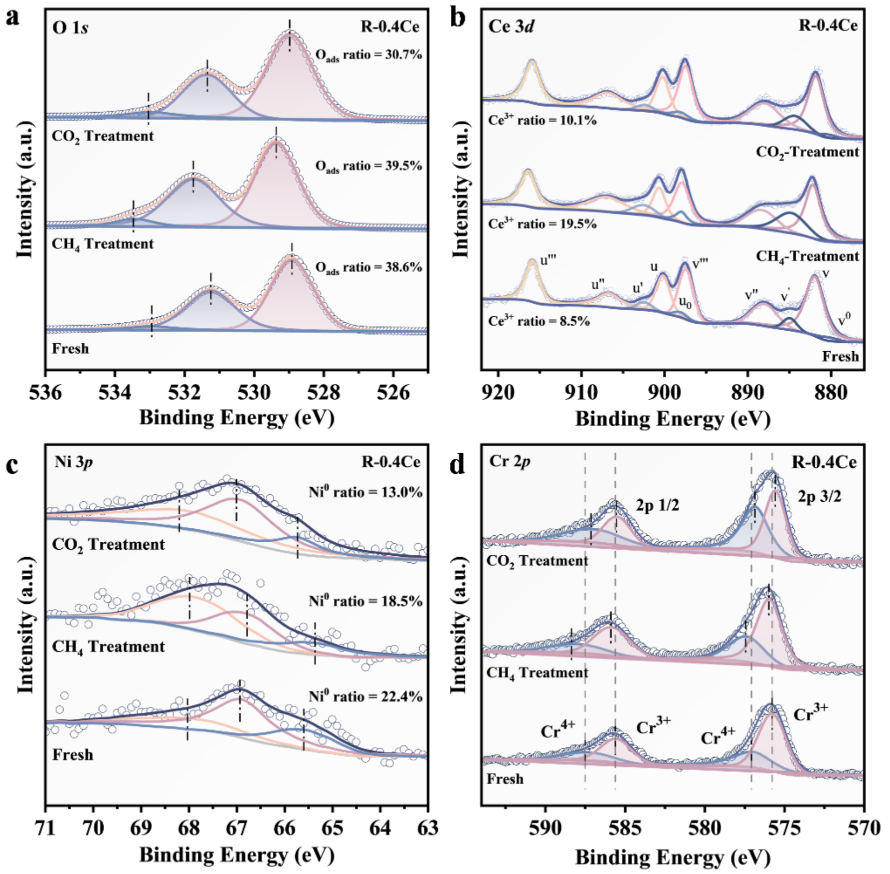

Figure S41. a O 1*s*, **b** Ce 3*d*, **c** Ni 3*p*, **d** Cr 2*p* XPS spectra of fresh 0.4Ce-$La_{0.97}Ni_{0.4}Cr_{0.6}O_3$, after CH_4 treatment, and after CO_2 treatment.

XPS in different atmosphere were applied to elucidate the alterations in the electronic structure and oxidation state at the catalyst interface in methane and carbon dioxide atmospheres, as shown in Fig. S39-S41, and Table S8. The oxygen vacancy content of the Ce-doped samples shows a slight increase following CH_4 treatment, which is then followed by a more pronounced decrease in the CO_2 atmosphere. In contrast, the proportion of the R-0Ce samples remains essentially unchanged. This indicates that the Ce-doped samples demonstrate higher lattice oxygen activity, which is involved in the C-H activation process. It can be filled by CO_2 adsorption activation, which in turn replenishes the oxygen vacancies. The Ce^{3+} ratio exhibits comparable alterations to those observed in the O vacancies, initially increased and then decreased.

The lattice oxygen in the surface CeO_{2-x} is also involved in the activation of C-H bonds, while the Ce^{3+}-O_v structure is responsible for the dissociation of CO_2[4]. In contrast to other samples, which exhibited an elevated Ni^0 content following CH_4 treatment, the Ni 3*p* peak of the R-0.4Ce sample demonstrated a reduction in its Ni^0 ratio, accompanied by a shift towards a lower binding energy. A substantial accumulation of carbon may cover the surface Ni^0 active site. Furthermore, an electron transfer occurs between the Ni and Cr atoms. Following the CH_4 treatment, the Ni-O bond is weakened and the Cr-O bond is strengthened, which facilitates Ni segregated to the surface under a reducing atmosphere. It is worthy of note that the R-0Ce sample exhibited a Cr^{6+} peak following CO_2 treatment, which was not observed in the Ce-doped sample. The 4f shell layer electrons in the Ce^{3+}/Ce^{4+} redox pair in surface CeO_{2-x} are more readily lost in an oxidizing atmosphere, thereby allowing Cr to gain electrons and preventing it from being oxidized to Cr^{6+}. The smaller radius of Cr^{6+} in comparison to other B-site elemental ions will result in an increase in the tolerance factor (t), which would lead to a decrease in the degree of lattice distortion. This would have a detrimental impact on the exsolution kinetics, as well as on the formation and transfer of oxygen vacancies[5]. Furthermore, the reduction of the high-valent Cr ion content enhances the environmental friendliness of the catalyst.

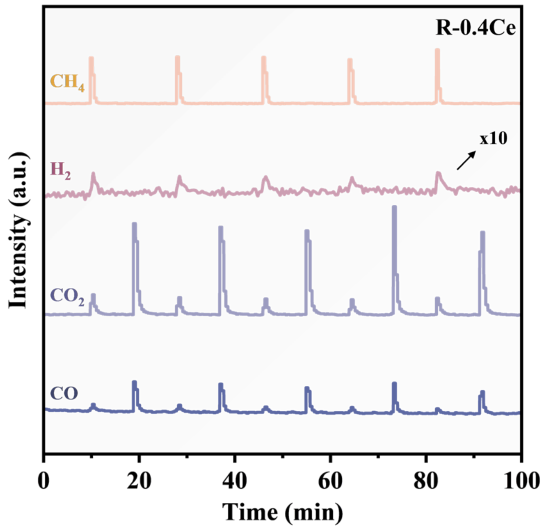

Figure S42. MS signal of CH_4/CO_2 alternately pulse experiments at 750 °C on 0.4Ce-$La_{0.97}Ni_{0.4}Cr_{0.6}O_3$.

Specifically, the CO_2 peak intensity of R-0.2Ce during CH_4 pulse is markedly higher than that of others, owing to the rich oxygen vacancy from surface CeO_{2-x} species and the satisfied lattice oxygen mobility induced by lattice distortion. Moreover, the H_2O peaks exhibit much obvious delay than H_2, which could be explained by the slow combination of H· formed by C-H dissociation and surface O species to generate water. The peak represents CO production during CO_2 pulses performs a gradual increase and subsequent stability trend. CO_2 can be adsorbed and undergo C=O bond-breaking reactions with surface oxygen vacancies, resulting in the production of CO with the achievement of oxygen vacancy replenishment. Moreover, CO may be also produced by a reverse Boudouard reaction, which suppresses the deposited carbon accumulation.

The product peak signal of the 0.4Ce sample is notably diminished in comparison to the remaining samples. This is attributed to the decrease in active sites (larger Ni nanoparticle size and a narrower density distribution characterized by TEM/SEM). Additionally, the surface of the perovskite phase is more extensively covered by CeO_{2-}

$_x$, and the reduction process gives rise to positively charged oxygen vacancies with a high affinity for the perovskite bulk-phase lattice oxygen. This phenomenon maintains the surface Ce oxidation state at a higher level with a reduced number of surface oxygen vacancies, consistent with the results of XPS analysis. Therefore, the activation capacity for CO_2 is also relatively low.

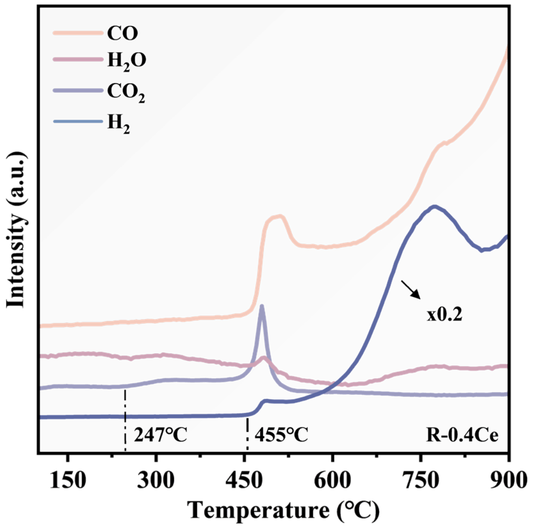

Figure S43. CH_4-TPSR of 0.4Ce-$La_{0.97}Ni_{0.4}Cr_{0.6}O_3$.

The abundant lattice oxygen present in the CeO_{2-x} on the perovskite surface permitted the complete oxidation of CH_4 at lower temperatures. Once the surface lattice oxygen was consumed, the lattice oxygen in bulk phase was gradually transferred to the surface at higher temperatures, resulting in the occurrence of partial oxidation of methane.

5. Characterizations of R-0Ce and R-0.2Ce after long-term DRM test

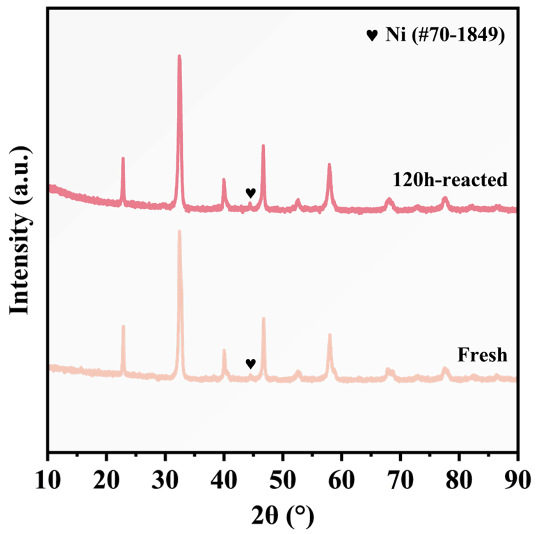

Figure S44. XRD pattern of fresh and 120h-reacted R-0Ce.

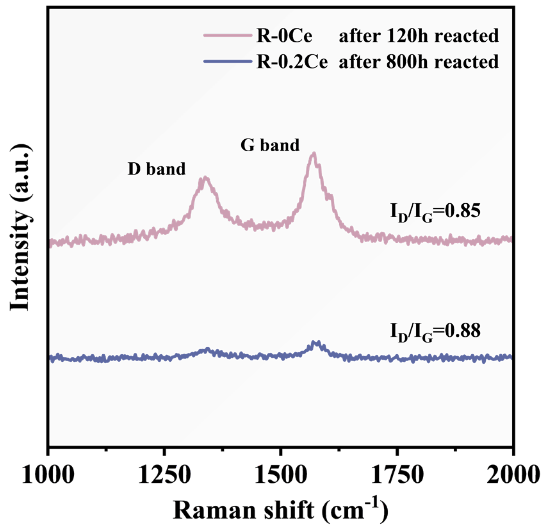

Figure S45. Raman spectra of 120h-reacted R-0Ce and 800h-reacted R-0.2Ce.

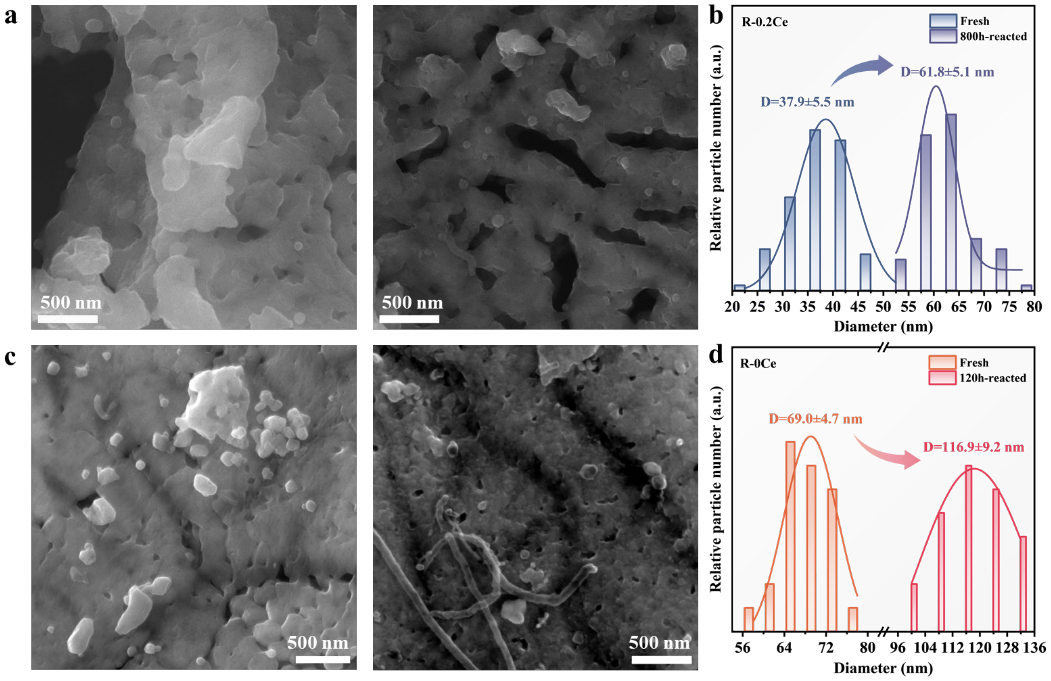

Figure S46. **a** SEM images of 800h-reacted R-0.2Ce. **b** Particle size of R-0.2Ce. **c** SEM images of 120h-reacted R-0 Ce. **d** Particle size of R-0Ce.

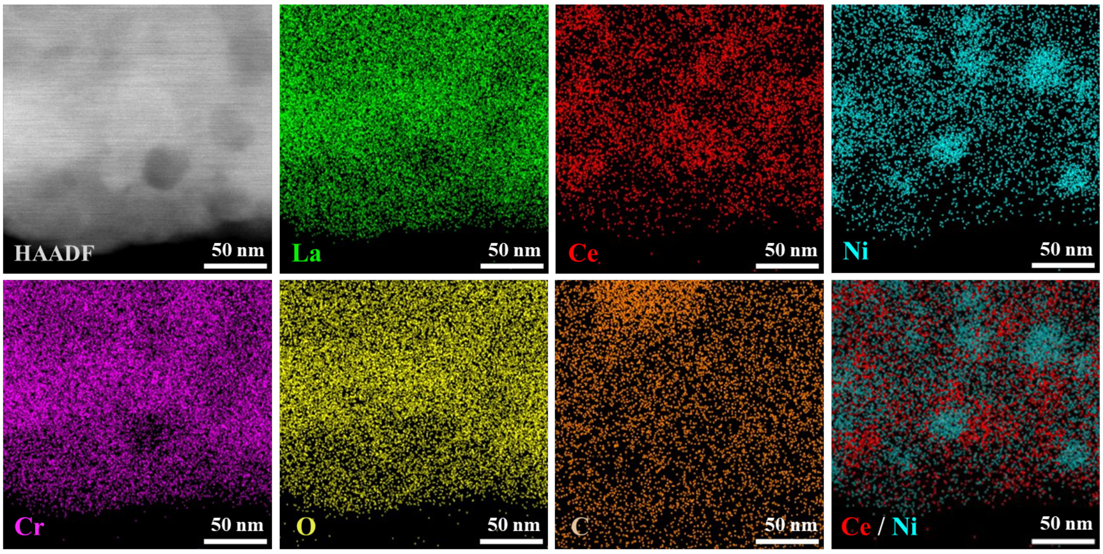

Figure S47. TEM images and EDS mapping of 800h-reacted R-0.2Ce.

The TEM images of 800h-reacted R-0.2Ce (as shown in Fig. S47) further shows that some of the nanoparticles still maintain a small particle size (<50 nm). In addition, it can be found that the distribution of Ce is mostly clustered near the exsolved Ni nanoparticles.

6. Reaction mechanism of DRM.

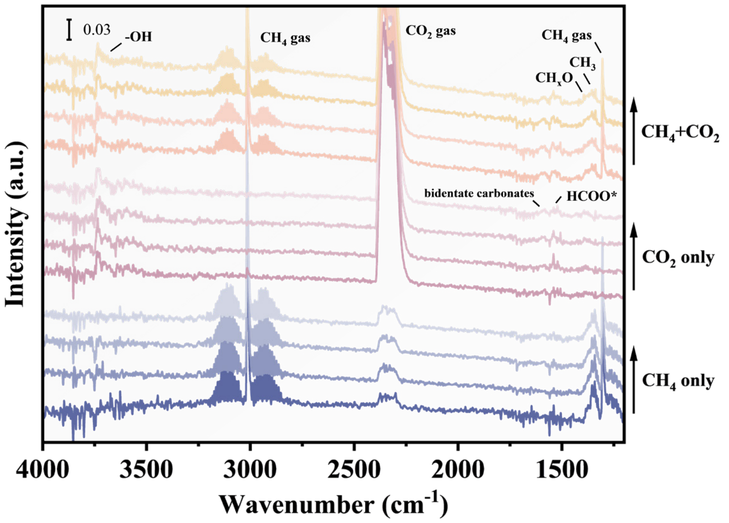

Figure S48. In-situ DRIFTS results of 0.4Ce-$La_{0.97}Ni_{0.4}Cr_{0.6}O_3$.

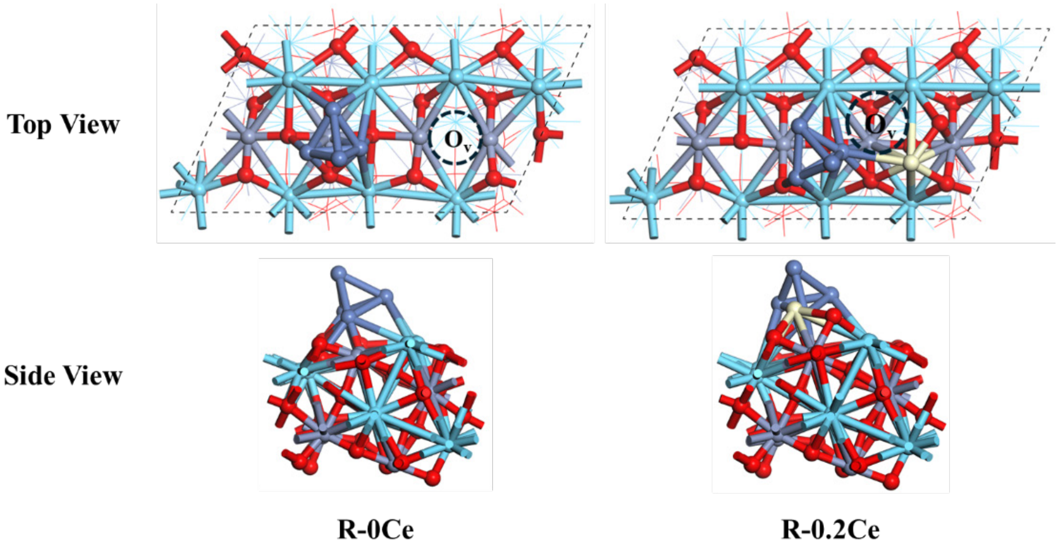

Figure S49. The structures of initial states and final states for CH_2 dissociation into CH^* or reacting with O to CH_2O^* on the R-0Ce and R-0.2Ce catalysts.

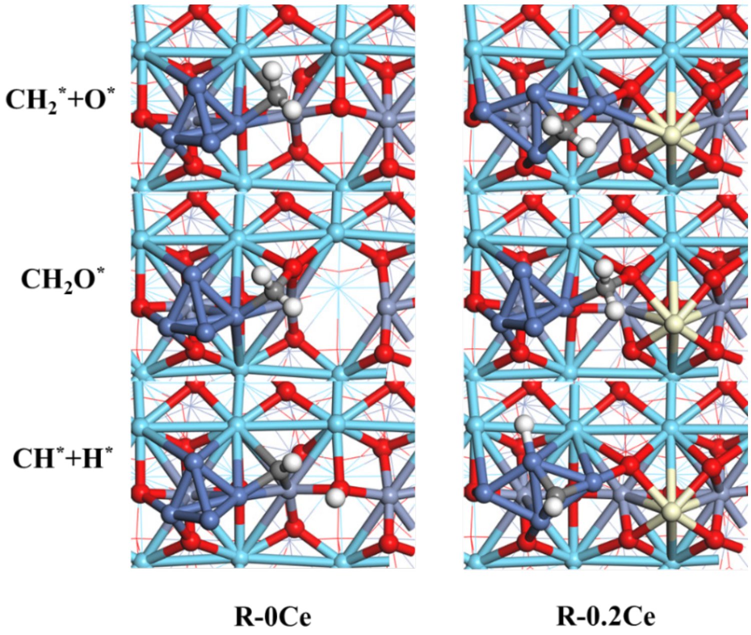

Figure S50. Surface morphology of R-0Ce and R-0.2Ce catalysts. Red, light blue, blue-gray, steel blue, and beige balls represent O, La, Cr, Ni, and Ce atoms, respectively.

Table S1. Comparison of the DRM performance with literature.

Catalysts	Active metals loading (wt %)	Reaction conditions	Specific activity (mmol·$g_{active\ metals}^{-1}$·s^{-1}) / Conversion (%)		H_2/CO	GHSV (mL·g_{cat}^{-1}·h^{-1})	T (°C)	TOS (h)	Coke formation rate (mmol·g_{cat}^{-1}·s^{-1})	Ref.
			CH_4	CO_2						
**0.2Ce-$La_{0.97}Ni_{0.4}Cr_{0.6}O_3$**	**7.76% Ni**	**CH_4:CO_2:N_2=1:1:1**	**1.38 / 87.4**	**1.49 / 92.9**	**0.92**	**30000**	**800**	**800**	**4.63E-07**	**This work**
**$LaNi_{0.4}Cr_{0.6}O_3$**	**8.55% Ni**	**CH_4:CO_2:N_2=1:1:1**	**1.16 / 79.7**	**1.27 / 87.9**	**0.89**	**30000**	**800**	**120**	**1.23E-05**	**This work**
$La(Co_{0.1}Ni_{0.9})_{0.5}Fe_{0.5}O_3$	12.03% Ni-Co	CH_4:CO_2=1:1	0.43 / 70.0	0.49 / 80.2	0.87	12000	750	30	6.17E-07	[7]
$La(Co_{0.3}Ni_{0.7})_{0.5}Fe_{0.5}O_3$	12.02% Ni-Co	CH_4:CO_2=1:1	0.43 / 70.2	0.50 / 80.0	0.88	12000	750	30	1.16E-06	[7]
$La_{0.9}Sr_{0.1}Ni_{0.5}Fe_{0.5}O_3$	12.60% Ni	CH_4:CO_2:He=1:1:1	0.27 / 47.0	0.40 / 55.0	/	18000	700	50	1.85E-05	[8]
$La_{0.9}Sr_{0.1}NiO_3$	23.60% Ni	CH_4:CO_2:He=1:1:1	0.22 / 69.9	0.26 / 70.0	/	18000	700	8	8.97E-04	[8]
$LaNi_{0.6}Mn_{0.4}O_3$	14.46% Ni	CH_4:CO_2:N_2=1:1:2	0.30 / 91.0	0.45 / 85.0	1.02	15000	750	10	7.16E-05	[9]
$LaNiO_3$	23.96% Ni	CH_4:CO_2:N_2=1:1:2	0.16 / 75.0	0.23 / 70.0	1.08	15000	750	10	3.77E-04	[9]

$LaFe_{0.8}Ni_{0.2}O_3$ [a]	4.82% Ni	CH_4:CO_2:He=1:1:18	0.35 / 75.0	0.39 / 85.0	0.78	36000	700	24	/	[10]
$LaFe_{0.8}Ni_{0.2}O_3$ [b]	4.82% Ni	CH_4:CO_2:He=1:1:18	0.32 / 70.0	0.38 / 82.0	0.73	36000	700	24	/	[10]
$PrBaFeCoO_{5+\delta}$	23.47% Co-Fe	CH_4:CO_2:N_2=1:1:3	0.04 / 13.0	0.09 / 28.0	0.35	30000	900	120	6.94E-06	[11]
$La_{0.9}Ca_{0.1}Ni_{0.5}Fe_{0.5}O_3$	12.53% Ni	CH_4:CO_2=1:1	1.06 / 72.0	1.14 / 77.0	/	30000	750	500	≈ 0	[12]
$La_{0.9}Ca_{0.1}Ni_{0.3}Fe_{0.7}O_3$	7.54% Ni	CH_4:CO_2=1:1	0.44 / 18.0	1.01 / 41.0	/	30000	750	130	8.90E-04	[12]
$La_{0.9}Ca_{0.1}NiO_3$	24.90% Ni	CH_4:CO_2=1:1	0.31 / 42.0	0.49 / 66.0	/	30000	750	40	/	[12]
$Pr_{0.5}Ba_{0.5}Mn_{0.8}Ni_{0.15}Fe_{0.05}O_3$	4.78% Ni-Fe	CH_4:CO_2:N_2=33:34:33	1.34 / 52.0	1.53 / 58.0	0.80	30000	800	260	4.71E-08	[13]
$Pr_{0.5}Ba_{0.5}Mn_{0.8}Ni_{0.2}O_3$	4.83% Ni	CH_4:CO_2:N_2=33:34:33	2.03 / 80.0	2.12 / 81.0	0.84	30000	800	135	1.03E-04	[13]
$La_{0.9}Sr_{0.1}Fe_{0.95}Ni_{0.05}O_3$	1.23% Ni	CH_4:CO_2:Ar=1.5:1:27.5	0.28 / 93.0	0.20 / 99.0	1.10	6000	900	20	1.74E-04	[14]
$Pr_{0.45}Ba_{0.45}Mn_{0.8}(Co_{1/3}Ni_{2/3})_{0.2}O_{3\pm\delta}$	5.14% Ni-Co	CH_4:CO_2:N_2=1:1:8	0.17 / 84.0	0.19 / 93.0	0.93	8570	850	50	/	[15]
$Pr_{0.5}Ba_{0.5}Mn_{0.85}Ni_{0.1}Fe_{0.045}Rh_{0.005}O_3$	3.67% Ni-Fe-Rh	CH_4:CO_2:N_2=33:34:33	2.80 / 70.0	3.35 / 81.0	0.84	36000	750	120	3.87E-07	[16]

$La_{0.8}Sm_{0.2}NiO_{3-\delta}$	23.67% Ni	CH_4:CO_2:Ar=3:2:5	0.25 / 48.0	0.42 / 81.0	/	40000	750	18	/	[17]
$La_{0.8}Ce_{0.2}NiO_{3-\delta}$	23.87% Ni	CH_4:CO_2:Ar=3:2:5	0.19 / 37.0	0.33 / 64.0	/	40000	750	18	/	[17]
$LaNiO_3$	23.96% Ni	CH_4: CO_2: N_2: He = 1.41:0.93:0.18:1	0.30 / 95.0	0.19 / 95.0	1.75	15000	850	50	1.39E-04	[18]
$La_2Ni_{0.8}Cu_{0.2}O_4$	14.87% Ni-Cu	CH_4:CO_2:N_2=1:1:2	0.22 / 88.0	0.23 / 93.0	0.91	12000	750	50	2.22E-05	[19]
$La_2Ni_{0.6}Cu_{0.4}O_4$	15.06% Ni-Cu	CH_4:CO_2:N_2=1:1:2	0.21 / 85.0	0.22 / 90.0	0.96	12000	750	50	2.23E-04	[19]
$LaFe_{0.7}Ni_{0.1}Co_{0.1}Cu_{0.05}Pd_{0.05}O_3$	8.23% Ni-Co-Cu-Pd	CH_4:CO_2:N_2=1:1:18	0.06 / 70.0	0.06 / 79.0	/	/	700	24	/	[20]
$LaFeNi_{0.05}Co_{0.05}O_3$	2.37% Ni-Co	CH_4:CO_2:N_2=1:1:18	0.22 / 78.0	0.21 / 85.0	/	/	800	24	/	[20]
$La_{1.8}Ba_{0.2}Ni_{0.9}Cu_{0.1}O_4$	14.77% Ni-Cu	CH_4:CO_2:He=1:1:3	0.93 / 46.0	1.21 / 60.0	0.90	120000	700	6	/	[21]
$PrBaMn_{1.7}Co_{0.1}Ni_{0.2}O_{5+\delta}$ + 15 wt % infiltration of Fe	18.20% Ni-Co-Fe	CH_4:CO_2:He=1:1:3	0.03	0.06	/	30000	750	350	≈ 0	[22]
$La_{0.6}Sr_{0.2}Cr_{0.85}Ni_{0.15}O_3$	4.37% Ni	CH_4:CO_2:N_2=9:9:2	0.34 / 88.3	0.30 / 88.0	/	3000	750	24	≈ 0	[23]
$La_{0.8}Ca_{0.2}Mn_{0.8}Ni_{0.2}O_{3\pm\delta}$	6.19% Ni	CH_4:CO_2:Ar=1:1:3	0.63 / 88.2	0.69 / 96.6	/	18000	800	500	3.47E-07	[24]

$La_2Ni_{0.6}Cu_{0.4}O_4$	8.75% Ni<br>6.32% Cu	$CH_4:CO_2:N_2$=1:1:2	0.21 / 88.0	0.23 / 92.0	/	12000	750	50	2.22E-05	[25]
$Ba_{0.9}Zr_{0.5}Ce_{0.2}Y_{0.2}Ni_{0.1}O_{3-\delta}$	2.18%<br>Ni	$CH_4:CO_2$ =1:1	1.79 / 71.0	/	/	9000	700	60	1.93E-06	[26]
$La_2Ce_2O_7$-$LaNiO_3$	5.00%<br>Ni	$CH_4:CO_2$ =1:1	1.96 / 89.0	2.03 / 91.0	0.63	18000	850	6	7.72E-05	[27]

a: 120 min reduction

b: 30 min reduction

* Coke formation rate calculated by the equation: *w* (weight loss%)/(12*3.6*TOS(h))

Table S2. The ICP-OES results of fresh samples.

Sample	Nominal loading (wt.%)				Actual loading (wt.%)			
	La	Ce	Ni	Cr	La	Ce	Ni	Cr
$LaCrO_3$	58.16	/	/	21.76	56.88	/	/	21.86
$LaNi_{0.4}Cr_{0.6}O_3$	57.53	/	9.72	12.92	56.93	/	8.551	12.08
$0.1Ce\text{-}La_{0.97}Ni_{0.4}Cr_{0.6}O_3$	52.91	5.50	9.22	12.25	51.78	4.66	8.31	11.52
$0.2Ce\text{-}La_{0.97}Ni_{0.4}Cr_{0.6}O_3$	49.55	10.30	8.64	11.48	49.10	9.03	7.76	11.02
$0.3Ce\text{-}La_{0.97}Ni_{0.4}Cr_{0.6}O_3$	46.61	14.54	8.12	10.79	46.44	12.86	7.47	10.04
$0.4Ce\text{-}La_{0.97}Ni_{0.4}Cr_{0.6}O_3$	43.99	18.29	7.67	10.19	43.00	16.99	6.71	9.27

The ICP-OES results demonstrate that the mass fractions of all elements present in the prepared catalyst samples are in close agreement with the theoretical values, as shown in Table S1.

Table S3. The XRD Rietveld refinement results, unit cell parameters and oxygen deficiency of 0.2Ce sample.

sample	Space group / Lattice parameters										
	Pnma					R -3c					
	a (Å)	b (Å)	c (Å)	volume (Å^3)	δ_{ex}	a (Å)	b (Å)	c (Å)	volume (Å^3)	δ_{ex}	Ni^0 wt.%
0.2Ce-$La_{0.97}Ni_4Cr_6O_3$ After calcination	5.468	7.753	5.509	233.584	0	/	/	/	/	/	/
0.2Ce-$La_{0.97}Ni_xCr_{1-x}O_{3-\delta}$ After reduction	5.474	7.783	5.517	235.082	0.08	5.51817	5.51817	13.31620	351.158	0.12	6.79%
$LaCrO_3$	5.486	7.768	5.525	235.437	0	5.53049	5.53049	13.35440	353.738	0	/

The δ_{ex} represents the non-stoichiometric oxygen, as shown in Table S2.

Table S4. The XRD Rietveld refinement results, unit cell parameters and oxygen deficiency of 0Ce sample.

sample	Space group / Lattice parameters										
	Pnma					R -3c					
	a (Å)	b (Å)	c (Å)	volume ($Å^3$)	δ_{ex}	a (Å)	b (Å)	c (Å)	volume ($Å^3$)	δ_{ex}	Ni^0 wt. %
$LaNi_{0.4}Cr_6O_3$ After calcination	5.470	7.760	5.510	233.915	0	/	/	/	/	/	/
$LaNi_xCr_{1-x}O_{3-\delta}$ After reduction	5.490	7.768	5.518	235.341	0.06	5.51905	5.51905	13.30180	350.889	0.07	3.51%
$LaCrO_3$	5.486	7.768	5.525	235.437	0	5.53049	5.53049	13.35440	353.738	0	/

Table S5. Results of the fitting of the EXAFS curves for Ni K-edge EXAFS. The experimental data (k-range: 2.5-14.0 Å^{-1}, R-range: 1.0-3.3 Å) were fitted in R-space (k-weighting=3).

Samples	Path	R (Å) ^{a}	Coordination number	σ^2 (10^{-3}Å^2) ^{b}	ΔE_0 (eV) ^{c}	R-factor (%)
Ni-foil	Ni-Ni	2.48±0.002	12	0.006	6.07±0.41	0.19
NiO	Ni-O	2.07±0.010	7.01±1.12	0.008	-3.09±0.81	0.64
	Ni-Ni	2.96±0.006	13.71±1.52	0.008		
$LaNi_{0.4}Cr_{0.6}O_3$	Ni-O_1	1.89±0.043	0.96±0.14	0.006	-4.28±0.72	0.81
	Ni-O_2	2.02±0.067	1.61±0.28	0.001		
	Ni-Ni/Cr	2.49±0.001	3.28±0.37	0.001		
	Ni-La	3.33±0.055	1.66±0.31	0.007		
0.2Ce-$La_{0.97}Ni_{0.4}Cr_{0.6}O_3$	Ni-O_1	1.92±0.006	0.63±0.33	0.005	-6.01±0.99	0.49
	Ni-O_2	2.06±0.038	1.09±0.43	0.001		
	Ni-Ni/Cr	2.49±0.006	3.94±0.34	0.005		
	Ni-La	3.28±0.028	1.74±0.65	0.008		

^{a} R: bond length. ^{b} σ^2: Debye-Waller factor. ^{c} ΔE_0: the inner potential correction.

Table S6. Summary of the linear combination fittings (LCF) from the XANES results

Standard	Weight (sum to 1)	R-factor	Chi-square
$LaNi_{0.4}Cr_{0.6}O_3$	0.907 ± 0.003	0.01%	0.002
Ni	0.093 ± 0.003		

Table S7. Summary of XPS Characteristics of reduced samples.

Catalyst	Binding Energy (eV)				Binding Energy (eV)			Binding Energy (eV)			Binding Energy (eV)			
	O_{lat}	O_{ads}	H_2O	O_{ads}/O_{lat}	Ce^{3+}	Ce^{4+}	$Ce^{3+}/(Ce^{3+}+Ce^{4+})$	Cr(III)	Cr(IV)	Cr(III) ratio	Ni^0	Ni^{2+}	Ni^{3+}	Ni^0 ratio
$LaNi_{0.4}Cr_{0.6}O_3$	529.0	531.3	533.0	46.1%	/	/	/	575.5, 585.3	576.7, 586.9	49.5%	66.1	67.1	68.1	22.4%
0.1Ce-$La_{0.97}Ni_{0.4}Cr_{0.6}O_3$	528.9	531.3	533.0	45.9%	880.2, 885.3, 898.2, 902.5	881.9, 888.1, 897.4, 900.2, 906.5, 915.8	19.5%	575.6, 585.3	576.7, 586.9	47.3%	66.0	67.0	68.1	24.5%
0.2Ce-$La_{0.97}Ni_{0.4}Cr_{0.6}O_3$	528.9	531.2	533.0	44.1%	880.2, 885.2, 898.2, 902.6	882.1, 888.1, 897.4, 900.2, 906.5, 916.0	18.6%	575.6, 585.4	576.8, 587.0	46.1%	65.9	67.0	68.0	27.4%
0.3Ce-$La_{0.97}Ni_{0.4}Cr_{0.6}O_3$	528.9	531.3	533.0	40.0%	880.2, 885.0, 898.1, 902.5	881.9, 888.0, 897.4, 900.2, 906.6, 916.0	16.4%	575.7, 585.5	577.0, 587.2	44.6%	65.9	67.0	68.0	25.8%
0.4Ce-$La_{0.97}Ni_{0.4}Cr_{0.6}O_3$	529.0	531.3	533.0	38.6%	880.3, 885.0, 898.1, 902.5	882.0, 888.0, 897.4, 900.2, 906.6, 915.9	8.5%	575.8, 585.6	576.9, 587.2	44.1%	65.5	66.9	67.9	22.4%

Table S8. Summary of CH_4, CO_2 treated XPS Characteristics.

Catalyst	Binding Energy (eV)				Binding Energy (eV)			Binding Energy (eV)			Binding Energy (eV)			
	O_{lat}	O_{ads}	H_2O	O_{ads}/O_{lat}	Ce^{3+}	Ce^{4+}	Ce^{3+} ratio	Cr^{3+}	Cr^{4+}	Cr^{3+} ratio	Ni^{0}	Ni^{2+}	Ni^{3+}	Ni^{0} ratio
$LaNi_{0.4}Cr_{0.6}O_3$	529.0	531.3	533.0	46.1%	/	/	/	575.5, 585.3	576.5, 586.7	38.5%	66.1	67.1	68.1	22.4 %
CH_4 Treatment	529.4	531.4	533.2	44.8%	/	/	/	575.8, 585.8	577.0, 587.0	43.9%	65.9	66.9	68.2	24.0 %
CO_2 Treatment	529.0	531.3	533.1	43.1%	/	/	/	575.2, 585.1	576.4, 586.3 578.9, 588.9 (Cr^{6+})	24.0%	66.1	66.9	68.2	23.8 %
0.2Ce-$La_{0.97}Ni_{0.4}Cr_{0.6}O_3$	528.9	531.2	533.0	44.1%	880.2, 885.2, 898.2, 902.6	882.1, 888.1, 897.4, 900.2, 906.5, 916.0	18.6%	575.6, 585.4	576.8, 587.0	55.5%	65.9	67.0	68.0	27.4 %
CH_4 Treatment	529.4	531.6	533.5	44.5%	881.0, 885.8, 898.4, 903.4	882.6, 888.9, 898.0, 900.9, 907.5, 916.5	26.7%	575.9, 585.8	577.0, 587.2	55.2%	65.8	66.8	68.0	33.4 %
CO_2 Treatment	529.1	531.5	533.5	38.4%	880.2, 885.3, 898.0, 902.6	882.1, 888.1, 897.8, 900.4, 906.5, 916.1	20.3%	575.5, 585.4	576.6, 586.8	42.9%	65.9	66.9	68.1	28.5 %

0.4Ce-$La_{0.97}Ni_{0.4}Cr_{0.6}O_3$	529.0	531.3	533.0	38.6%	880.3, 885.0, 898.1, 902.5	882.0, 888.0, 897.4, 900.2, 906.6, 915.9	8.5%	575.8, 585.6	577.0, 587.3	64.8%	65.5	66.9	67.9	22.4 %
CH_4 Treatment	529.4	531.7	533.4	39.5%	880.3, 884.8, 898.0, 902.6	882.2, 888.3, 897.9, 900.7, 906.9, 916.4	19.5%	576.0, 585.8	577.4, 588.0	66.8%	65.4	66.8	67.8	18.5 %
CO_2 Treatment	529.0	531.4	533.0	30.7%	880.3, 884.4, 898.1, 902.4	881.8, 888.0, 897.5, 900.3, 906.8, 915.9	10.1%	575.6, 585.5	576.8, 587.0	49.8%	65.7	66.9	68.0	13.3 %

Table S9. Bader charge of Ni atoms (*e*), as well as the total Bader chafe of Ni_4 cluster on the R-0Ce and R-0.2Ce catalysts.

**Bader charge**	**R-0Ce**	**R-0.2Ce**
Ni_1	0.21	0.03
Ni_2	0.35	0.19
Ni_3	-0.47	-0.48
Ni_4	-0.12	-0.17
Total	-0.03	-0.43

Table S10. Bader charge of Ce atoms (*e*), as well as the total Bader chafe of CeO_{2-x} cluster on the R-0Ce and R-0.2Ce catalysts.

Bader charge	CeO_{2-x}	R-0.2Ce
Ce	1.15	1.87
O	-1.04	-1.31
Total	0.11	0.56

Table S11. Detailed list of abbreviations.

Full name	Abbreviations
Calcined $LaNi_{0.4}Cr_{0.6}O_3$	C-0Ce
Calcined 0.1Ce-$La_{0.97}Ni_{0.4}Cr_{0.6}O_3$	C-0.1Ce
Calcined 0.2Ce-$La_{0.97}Ni_{0.4}Cr_{0.6}O_3$	C-0.2Ce
Calcined 0.3Ce-$La_{0.97}Ni_{0.4}Cr_{0.6}O_3$	C-0.3Ce
Calcined 0.4Ce-$La_{0.97}Ni_{0.4}Cr_{0.6}O_3$	C-0.4Ce
Reduced $LaNi_{0.4}Cr_{0.6}O_3$	R-0Ce
Reduced 0.1Ce-$La_{0.97}Ni_{0.4}Cr_{0.6}O_3$	R-0.1Ce
Reduced 0.2Ce-$La_{0.97}Ni_{0.4}Cr_{0.6}O_3$	R-0.2Ce
Reduced 0.3Ce-$La_{0.97}Ni_{0.4}Cr_{0.6}O_3$	R-0.3Ce
Reduced 0.4Ce-$La_{0.97}Ni_{0.4}Cr_{0.6}O_3$	R-0.4Ce
Dry reforming of methane	DRM
Reverse water-gas shift	rWGS
Weight hourly space velocity	WHSV
Inductively coupled plasma optical emission spectroscopy	ICP-OES
X-ray diffractometer	XRD
Scanning electron microscopy	SEM
Energy dispersive spectroscopy	EDS
transmission electron microscopy	TEM
High-angle annular dark-field scanning transmission electron microscopy	HAADF-STEM
Atomic force microscope	AFM
X-ray photoelectron spectroscopy	XPS
Electron paramagnetic resonance	EPR
In-situ diffuse reflectance infrared fourier transform spectroscopy	In-situ DRIFTS
The X-ray absorption fine structure spectra	XAS
Density functional theory	DFT
The temperature-programmed reaction of methane	CH_4-TPR

Supplementary References

1. Cybulski A. Catalytic wet air oxidation are monolithic catalysts and reactors feasible? Ind. Eng. Chem. Res. **46**, 4007-33 (2007).
2. Bhattar S, et al. A review on dry reforming of methane over perovskite derived catalysts. Catal. Today. **365**, 2-23 (2021).
3. Zhang R, et al. Tailoring catalytic and oxygen release capability in $LaFe_{1-x}Ni_xO_3$ to intensify chemical looping reactions at medium temperatures. ACS Catal. **14(10)**, 7771-87 (2024).
4. Wang H, et al. Facilitating the dry reforming of methane with interfacial synergistic catalysis in an $Ir@CeO_{2-x}$ catalyst. Nat Commun. **15(1)**, 3765 (2024).
5. Sun Z, et al. Recent advances in exsolved perovskite oxide construction: exsolution theory, modulation, challenges, and prospects. J. Mater. Chem. A. **11(34)**, 17961-76 (2023).
6. Kim Y H, et al. Shape-shifting nanoparticles on a perovskite oxide for highly stable and active heterogeneous catalysis. Chem. Eng. J. **441**, 1 (2022).
7. Wang H, et al. Dry reforming of methane over bimetallic Ni-Co catalyst prepared from $La(Co_xNi_{1-x})_{0.5}Fe_{0.5}O_3$ perovskite precursor: Catalytic activity and coking resistance. Appl. Catal., B. **245**, 302-13 (2019).
8. Das S, et al. Effect of partial Fe substitution in $La_{0.9}Sr_{0.1}NiO_3$ perovskite-derived catalysts on the reaction mechanism of methane dry reforming. ACS Catal. **10(21)**, 12466-86 (2020).
9. Shahnazi A, et al. Improving the catalytic performance of $LaNiO_3$ perovskite by manganese substitution via ultrasonic spray pyrolysis for dry reforming of methane. J. CO_2 Util. **45**, 101455 (2021).
10. Shah S, et al. Dynamic tracking of NiFe smart catalysts using in situ x-ray absorption spectroscopy for the dry methane reforming reaction. ACS Catal. **13(6)**, 3990-4002 (2023).
11. Managutti P B, et al. Exsolution of Co-Fe alloy nanoparticles on the $PrBaFeCoO_{5+delta}$ layered perovskite monitored by neutron powder diffraction and catalytic effect on dry reforming of methan. ACS Appl. Mater. Interfaces. **15(19)**, 23040-50 (2023).
12. Oh J, et al. Precise modulation of triple-phase boundaries towards a highly functional exsolved catalyst for dry reforming of methane under a dilution-free system. Angew. Chem., Int. Ed. **61(33)**, e202204990 (2022).
13. Yao X, et al. Atypical stability of exsolved Ni-Fe alloy nanoparticles on double layered perovskite for CO_2 dry reforming of methane. Appl. Catal., B. **328(5)**, 122479 (2023).
14. Romay M, et al. Unravelling the effectiveness of the small partial substitution of Fe by Ni in $La_{0.9}Sr_{0.1}Fe_{1-x}Ni_xO_3$ perovskites to improve their performance in dry reforming of methane. Chem. Eng. J. **496(15)**, 154039 (2024).
15. Cao X, et al. Surface decomposition of doped $PrBaMn_2O_{5+\delta}$ induced by in situ nanoparticle

exsolution: quantitative characterization and catalytic effect in methane dry reforming reaction. Chem. Mater. **34(23)**, 10484-94 (2022).

16. Yao X, et al. Enlarging the three-phase boundary to raise CO_2/CH_4 conversions on exsolved Ni–Fe alloy perovskite catalysts by minimal Rh doping. ACS Catal. **14(8)**, 5639-53 (2024).
17. Georgiadis A G, et al. An experimental and theoretical approach for the biogas dry reforming reaction using perovskite-derived $La_{0.8}X_{0.2}NiO_{3-\delta}$ catalysts (X = Sm, Pr, Ce). Renewable Energy. **227**, 120511 (2024).
18. Moogi S, et al. Influence of catalyst synthesis methods on anti-coking strength of perovskites derived catalysts in biogas dry reforming for syngas production. Chem. Eng. J. **437(1)**, 135348 (2022).
19. Cao D, et al. Dry reforming of methane by La_2NiO_4 perovskite oxide: B-site substitution improving reactivity and stability. Chem. Eng. J. **482(15)**, 148701 (2024).
20. Najimu M, et al. Co-exsolution of Ni-based alloy catalysts for the valorization of carbon dioxide and methane. Acc. Chem. Res. **56(22)**, 3132-41 (2023).
21. Bekheet M F, et al. Steering the methane dry reforming reactivity of Ni/La_2O_3 catalysts by controlled in situ decomposition of doped La_2NiO_4 precursor structures. ACS Catal. **11(1)**, 43-59 (2021).
22. Joo S, et al. Enhancing thermocatalytic activities by upshifting the d-band center of exsolved Co-Ni-Fe ternary alloy nanoparticles for the dry reforming of methane. Angew. Chem., Int. Ed. **60(29)**, 15912-15919 (2021).
23. Wei T, et al. CO_2 dry reforming of CH_4 with Sr and Ni co-doped $LaCrO_3$ perovskite catalysts. Appl. Surf. Sci. **506(15)**, 144699 (2020).
24. Sojung K, et al. Designing highly active and stable ni-exsolved $LaMnO_3$ perovskite catalysts for dry reforming of methane via Ca substitution. ACS Catal. **15(9)**, 6812-6815 (2025).
25. Dingshan C, et al. Dry reforming of methane by La_2NiO_4 perovskite oxide: B-site substitution improving reactivity and stability. Chem. Eng. J. **482(15)**, 148701 (2024).
26. Qiu P, et al. Enhanced catalytic activity for methane dry reforming of Ce-doped $Ba_{0.9}Zr_{0.7}Y_{0.2}Ni_{0.1}O_{3-\delta}$ perovskite catalyst. Mol. Catal. **549**, 113543 (2023).
27. Ramon A P, et al. In situ study of low-temperature dry reforming of methane over $La_2Ce_2O_7$ and $LaNiO_3$ mixed oxides. Appl. Catal., B. **315(15)**, 121528 (2022).
